# Benchmark Evaluation of Protein–Protein Interaction Prediction Algorithms

**DOI:** 10.3390/molecules27010041

**Published:** 2021-12-22

**Authors:** Brandan Dunham, Madhavi K. Ganapathiraju

**Affiliations:** Department of Biomedical Informatics, University of Pittsburgh, Pittsburgh, PA 15232, USA; brd86@pitt.edu

**Keywords:** protein–protein interactions, computational prediction, evaluation, interactome

## Abstract

Protein–protein interactions (PPIs) perform various functions and regulate processes throughout cells. Knowledge of the full network of PPIs is vital to biomedical research, but most of the PPIs are still unknown. As it is infeasible to discover all of them experimentally due to technical and resource limitations, computational prediction of PPIs is essential and accurately assessing the performance of algorithms is required before further application or translation. However, many published methods compose their evaluation datasets incorrectly, using a higher proportion of positive class data than occuring naturally, leading to exaggerated performance. We re-implemented various published algorithms and evaluated them on datasets with realistic data compositions and found that their performance is overstated in original publications; with several methods outperformed by our control models built on ‘illogical’ and random number features. We conclude that these methods are influenced by an over-characterization of some proteins in the literature and due to scale-free nature of PPI network and that they fail when tested on all possible protein pairs. Additionally, we found that sequence-only-based algorithms performed worse than those that employ functional and expression features. We present a benchmark evaluation of many published algorithms for PPI prediction. The source code of our implementations and the benchmark datasets created here are made available in open source.

## 1. Introduction

Proteins are large macromolecules that perform a variety of functions necessary for an organism. Biological processes are driven by multiple proteins creating a protein complex or a signaling cascade through biophysical interactions. Understanding which proteins interact with each other is an important first step towards understanding molecular mechanisms of biological functions and of diseases, and in the design of therapeutics. Knowledge of binary protein–protein interactions (PPIs) is also useful in conducting further research on blocking or enhancing different interactions to control biological processes and functions, and to design therapeutics. However, given that human genome contains approximately 20,000 protein-coding genes and therefore nearly 200 million unique protein pairs even without accounting for multiple isoforms of genes, resolving all pairs of interacting protein is not a trivial task.

Many experimental biology methods have been devised to find pairs of interacting proteins. Benchwork experiments like co-immunoprecipitation (co-IP) are resource-intensive and low-throughput, often leading to false negatives depending on experimental conditions, for example due to ineffective antibodies or due to the transient nature of PPIs [1]. In contrast, high throughput screens such as yeast two-hybrid (Y2H) and tandem affinity purification mass-spectrometry (TAP-MS), and more recently a sequencing approach called PROPER-seq [2] attempt to capture tens-to-hundreds of thousands of PPIs in a single experiment. In the past, statistical estimates suggested that Y2H had 25%–45% false positives, and difficulties in detecting PPIs for certain subsets of proteins such as membrane proteins [3]. In later studies, it was shown that extensive filtering techniques can be used to decrease the false positive rate, usually by running multiple screens or comparing them to other data sources to obtain a more precise subset of potential interactions [4]. In one recent approach, a high quality protein interaction network of 53,000 PPIs was produced using such an approach [4]. However, the overlap between PPIs identified by different large-scale techniques remains low, with each method producing large amounts of PPIs unverified by any other experiment [5].

Overall, most PPIs out of an estimated 500,000 to 3 million binary PPIs of the human interactome remain unknown [4,6,7,8], which motivates the development of computational approaches for PPI discovery. At minimum, computational approaches should identify an accurate subset of likely interacting protein pairs that that can subsequently be validated with experimental techniques as true PPIs, thereby eliminating much of the cost in testing millions of other pairs. A sizeable amount of literature has been published on the computational prediction of PPIs, especially in model organisms like yeast, suggesting their extrapolation to human interactome mapping. These methods represent pairs of proteins by features of amino acid composition, protein functional annotations, or gene co-expression, and develop machine learning models to classify each pair (a data instance) as interacting or non-interacting.

Amino acid sequence-based predictors make up most of the literature for computationally predicting proteome-wide PPIs, except for yeast, where other features have also been widely explored. Sequence-based predictors rely on features computed from amino acid sequences, their physicochemical properties or unigram or n-gram counts. Examples of such feature representations include auto covariance (AC), pseudo amino acid composition (PSEAAC), and conjoint triads (CT), all of which have been widely used for predicting PPIs [9,10,11,12,13,14,15,16,17,18]. Other approaches attempt to make a more direct usage of amino acid sequences, either by generating per protein features from PSSMs using PSI-Blast, or by encoding the amino acids into a numeric vector to use as the features [13,19,20,21,22,23]. Once calculated, these values can build a numeric vector of equal length for each protein, creating a representation of data typically used in machine learning.

Non-sequence-based predictors utilize functional, protein subcellular location, structural and other biological annotation and transcriptomic data to compute features (henceforth referred to as annotation-based predictors in contrast to sequence-based predictors). These features rely heavily on Gene Ontology (GO), structural domain databases, and gene expression databases to assemble the features of all protein pairs, although there may be missing values for many pairs. They compute features of protein pairs, including cooccurrence in subcellular locations, co-expression correlation across different experimental conditions or across tissues, and semantic similarity of ontology annotations as input to machine learning classification models [24,25,26,27,28]. Many previous works rely on not one but a collection of these annotations, creating a feature vector per protein pair [29,30]. These collection-based classification algorithms have built-in methodologies to handle missing values so that entire protein pairs are not discarded when they miss some features.

Given the large number of unknown PPIs in the human interactome and the potential role of computational methods in discovering them, we must assess how accurately they perform on ‘real-world’ application, and if proven successful, compare them against each other to identify the best methods. In their original publications, most predictors are claimed to be highly accurate, with several obtaining over 90% accuracy, and some obtaining accuracies as high as 95–98%. Logically, if multiple classifiers exist that can obtain such high accuracies, predicting the full interactome should be a trivial task. However, the evaluations that these algorithms have been put through do not seem to consider two important aspects of PPIs, namely, (i) the large number of known PPIs involving a few hub proteins, and (ii) the rarity of PPIs among all possible protein-pairs. Here, we seek to evaluate and benchmark the performance of these algorithms, taking these aspects into account.

The common evaluation methodology for most PPI prediction methods is to utilize a set of known PPIs from a public repository as positive-label instances, and to use randomly sampled protein pairs (excluding the pairs that are known to interact) in place of negative-label instances. Randomly chosen pairs are utilized due to a lack of known, non-interacting pairs from experimental methods, as a failure to identify a PPI in a biological experiment only suggests that a PPI was not observed under those experimental conditions and does not prove that the two proteins never interact. Therefore, failed experiments do not create negative-labeled data. This lack of ability to experimentally determine non-interacting protein pairs prevents the creation of a traditional gold standard negative dataset. However, taking into account that only 0.325–1.5% of protein pairs have PPIs, computational methods utilize randomly sampled protein pairs as negatively labeled data, with the majority of randomly selected pairs likely being truly non-interacting pairs.

Although it is estimated that there exist about 500,000 to 3 million PPIs out of a total of 200 million protein pairs in humans (0.325–1.5%), most algorithms are trained and tested on datasets containing 50% positive label data. This simple approach for assessing an individual algorithm’s real-world application is questionable. Additionally, the protein interactome is believed to be a small-world network [31]. Such networks have hubs, i.e., nodes that connect directly to many other nodes, or in this case, proteins with a large number of PPIs. For example, The Biological General Repository for Interaction Datasets (BioGRID) contains ~125,000 unique, non-self-interactions among ~14,500 proteins (represented by their gene names/symbols and not distinguishing isoforms; this aspect that proteins are referred to by their genes is applicable throughout this paper except where explicitly mentioned otherwise) [32]. This averages to around 17 interactions per protein; however, currently 360 proteins have more than a 100 PPIs each, with one protein, *APP*, involved in over 2000 PPIs. On the other hand, over 9000 proteins have 10 or fewer PPIs each. While positive class PPI data are from a scale-free network, randomly paired data (negative class) are sampled uniformly, and thus have a significantly different distribution of proteins. This can lead to biasing problems in machine learning, where a single protein appears far more times in the positive dataset, allowing machine learning algorithms to simply predict pairs containing such proteins to be of positive labels, generating a high accuracy on the test datasets from corresponding distributions. Past experiments in this field have suggested similar findings, with works by Yu as well as Park and Marcotte suggesting that many prediction algorithms primarily predict bias in underlying datasets [33,34].

In addition to dataset creation, evaluation metrics also play a role in correctly assessing whether an algorithm can make good predictions on real data. In evaluating classification algorithms for rare category data, accuracy and AUC are not suitable methods, and precision-recall (P-R) curves are recommended [35]. In biological and clinical domains, where the natural distribution of class labels may be highly imbalanced, a P-R curve provides more reliable information and distinguishes models that are practical for real world applications, whereas AUC may misleadingly convey more impressive accuracy than are realistically achievable on the rare category that is of interest (e.g., an interacting protein-pair or the presence of a disease) [36]. However, most published works have used AUC/accuracy metrics. Both the aspects of data composition and evaluation metrics were taken into account by Qi et al. in the seminal paper on proteome-wide human PPI prediction (i.e., when a protein is compared against all the proteins) for membrane receptor proteins [29]. They set their test data at 1:1000 positive to negative instances and evaluated with a P-R curve. Our own method, called High-Precision Protein–Protein Interaction Prediction (HiPPIP) for human interactome prediction also was evaluated on the lines of Qi et al.’s, both in terms of rareness of positive class and using a P-R curve [37]. Additionally, the HiPPIP method was also evaluated for its ability to predict proteome-wide PPIs, by computing the cumulative number of true-positives versus the increasing number of false-positives (note that in this scenario of proteome-wide evaluation (i.e., where a hub protein is paired with every other protein in the human proteome), the unlabeled data may not all be false-positives, and may indeed me true positives that are currently unknown) on hub proteins with the reasonable assumption that many, if not all, of their PPIs are known; this metric was used as a reasonable approximation of the number of accurate predictions produced by HiPPIP.

In this study, we evaluate different PPI prediction algorithms to determine how well they perform on realistically proportioned datasets. We first implement various algorithms, using similar feature sets and classification models to those described in the original publications. Where possible, we download the datasets used by these algorithms to test whether our implementations produce similar results. Next, we created six new evaluation datasets containing three proportions of positive label instances (50%, 10% and 0.3%) and two sampling methods (sampled randomly from the full list of proteins as is commonly done in literature, henceforth known as Full), and using a held out set of proteins for evaluation (referred to as Held-Out, known as C3 data in Park and Marcotte’s prior work [33]). Finally, we also created control models to compare these predictors against, by using *illogical* features (e.g., frequency of the proteins in the dataset or random vectors to represent the proteins) with simple, naïve classifiers. These types of predictions do not consider the pairwise compatibility of two proteins, and simply predict PPIs based on the distinct distributions of proteins in positive and negative classes in the datasets. This allows us to determine how well the algorithms perform relative to these illogical features.

## 2. Methods

### 2.1. Data

Datasets used to validate the implementation of various sequence-based interaction predictors were downloaded from previous works. The datasets were adjusted from their original formats where necessary to include only proteins with a sequence length greater than 30 amino acids, as several algorithms have this requirement. Datasets which provided only protein names instead of the sequences had protein sequence data downloaded from UniProt (21 June 2021, 25 June 2021, 6 August 2021, 17 August 2021), with protein pairs removed if the names no longer mapped to valid proteins.

For prediction methods that employ protein annotations, the data were not downloaded from original sources for validation because most methods do not provide their datasets, and the availability of protein annotations such as domains, functions, expression and localizations increases over time. Some annotation databases are no longer available (e.g., InterDom), while others have increased their number of annotations since original publications; thus, we cannot try to match our results from these methods against the original publications. We treat our implementations of these as methods to be inspired by the original publications rather than exact reproductions. To create the necessary features, the Gene Ontology was download from the Gene Ontology Consortium (28 March 2021), and GO annotations of proteins were downloaded from the Gene Ontology Annotation Database (16 February 2021) [38,39]. Domain features were downloaded from InterPro (27 March 2021), Prosite (17 September 2021), and Pfam (17 September 2021) to compute domain and family-related features [40,41,42]. When annotations are provided using UniProt Identifiers, a union of all features assigned to a given Entrez Gene ID was used as features for a given protein [43].

Known PPI data were downloaded from the BioGRID (v4.4.198, compiled 25 May 2021). Only direct biophysical protein interactions, encoded by Proteomics Standards Initiative—Molecular Interaction (PSI-MI) ontology identifier MI:0407 and its descendants, were included. After filtering out pairs containing non-human proteins, self-interactions, and protein-RNA bindings, the Entrez Gene IDs provided by BioGRID were then mapped to UniProt IDs [43,44]. To account for Entrez Gene IDs mapping to multiple UniProt identifiers, the longest amino acid sequence was assigned to the gene. To ensure compliance with various sequence-based algorithms, protein sequences less than or equal to 30 amino acids were removed. A total of 123,626 unique interactions among 14,678 proteins remained as positively labeled protein pairs (i.e., PPIs). A total of 19,115 proteins with UniProt ID to Entrez Gene ID mapping that met the minimum sequence length were used to represent the full set of human proteins, with random pairs drawn from this dataset to use as negative labeled protein pairs (i.e., non-interacting protein pairs) (see Supplementary File S1 for details).

Training and testing datasets were created using two methodologies: In the first, a random set of non-overlapping interacting pairs and non-interacting pairs are sampled for training and testing from the full set of all possible proteins (Full). This method is widely used in the literature. We do not take any other precautions when randomly sampling non-interacting pairs, such as selecting proteins in different subcellular locations, as this could induce a bias towards the type of protein pairs chosen and creates a separate bias by limiting the number of proteins available to use in the non-interacting dataset. The second methodology is based on the work of Park and Marcotte, where some proteins are held out and used exclusively in the test dataset (Held Out) [33]. For this dataset creation method, we separated the proteins into 6 equal sized bins and hold out either 1 or 2 bins to create the test data. No pair using a protein from these bins is used in training data. Training data is created from bins excluding those held-out to create test data.

Multiple datasets were created for testing with different percentage compositions of positively labeled protein pairs (known interactions). Specifically, training sets were created with 50% and 20% positive data, while test sets were created with 50%, 10%, and 0.3% positive data. Models generated to test both the 10% and 0.3% positive test datasets used the 20% positive data for training, while 50% positive test datasets utilized 50% positive train datasets for training. For data based on random pairs, the five 50% positive train and test sets were created using stratified cross validation on a single set of 125,000 protein pairs. Data for the 20% training set, and 10% and 0.3% positive test sets, were chosen randomly from all pairs while ensuring that no test data overlapped with the training data. For the second method, namely by holding out proteins used in test data, training and test sets of the same ratios as mentioned earlier were created for each of the 21 combinations of holding out 1 or 2 of the 6 bins. Again, the 10% positive and 0.3% positive test sets shared the same training sets with 20% positive data.

### 2.2. Feature Computation

We replicated various sequence-based features as described in prior publications. Some, such as auto covariance and conjoint triads are used in multiple papers we reimplement, whereas others such as the Weighted-Skip Sequential Conjoint Triad method are used in only a single paper utilized in our recreations. While most methods listed can be computed from the sequences of the proteins in the pair, two of the listed methods required usage of other proteins’ sequence data. Skip-gram models compute the similarity of words, or in this case, amino acids, by training a neural network with neighboring words to create word embeddings [45]. For protein sequence features, we trained the skip-gram models using all protein sequences related to the organism under study. The second method, PSSMs (i.e., protein sequence similarity matrices), were computed using PSI-BLAST over UniProt’s SwissProt protein sequence database, using 3 iterations and a significance value of 0.001 [46]. If no hits were found, sequences were encoded using BLOSUM62 values. For features relying on physicochemical properties (e.g., hydrophobicity, hydrophilicity, polarity, charge, solvent-accessible surface area, etc.), we employed commonly used amino acid property values unless otherwise specified (see Supplementary File S2 for details). Finally, where possible, we normalized values produced by different feature computations to map to a low range of values, preferably between 0 and 1, or −1 and 1 (see Supplementary File S2 for details).

For annotation-based features, we primarily focused on pairs of domains and GO annotations appearing in known interactions, as well as semantic similarity calculations on GO annotations. As proteins commonly have multiple GO annotations and domains, many algorithms compute these features as a grid of scores between all possible pairs of annotations between a pair of proteins and convert the grid to a single score through an aggregation, such as average, max, sum, product, or the best matching average (see Supplementary File S3 for details). All protein pairs where the necessary features did not exist were scored as zero to ensure that we could train and test using the same datasets used for sequence-based prediction testing.

### 2.3. Model Construction

Classification models were constructed using Python (version 3.8.5) libraries Scikit-Learn, PyTorch, LightGBM, and ThunderSVM, or an independently created Rotation Forest library [47,48,49,50,51]. While methods in the selected publications have used other programming languages and/or libraries, these should be capable of reproducing similar results.

Models for sequence-based features were constructed to match published methods as closely as possible. For some models, such as support vector machines (SVMs), which are highly sensitive to different scales between features, features were scaled using min-max or standard scaling. Learning rates for neural networks implemented in PyTorch were adjusted to obtain similar results as reported in prior works, and all models were fitted with a decaying learning rate to ensure that the model would conclude quickly when learning had plateaued, ensuring quick running times on larger datasets. For publications that do not clearly state hyperparameters or other exact details used to construct models, we utilized settings that seemed similar to concepts in the referenced publication or other related publications, executed quickly, and performed comparably to reported results in the publications on their datasets. A total of 36 algorithms utilizing random forests, rotation forests, boosting, neural networks, and support vector machines were implemented based on previously published literature (see Supplementary Files S4 and S5 for details related to hyperparameters and any changes between previous work and our implementations).

For annotation-based features, a total of 6 models were created using domain and GO data. Two of these models, based on the works of Chen et al. and Maetschke et al., use large binary/ternary features to represent protein pairs. Chen et al. utilizes a pairwise sum of binary features containing which domains existed in each protein [26]. Maetschke et al. generated binary feature vectors from GO annotations, with non-zero values representing all annotations up to the lowest common ancestor (LCA) common to both proteins [28]. The final 4 annotation-based models used significantly smaller feature representations for each protein pair. Guo et al. and Zhang et al. each developed models relying on semantic similarity using maximum aggregation. Guo et al. relied on a single metric per ontology and a logistic regression model, while Zhang used 10 different semantic similarity measurements to train an SVM [24,25]. Zhang et al. used a score based on different domain databases to predict protein interactions, taking the maximum score across the databases per domain pair and using product aggregation to create a final score [27]. As we are not using pre-computed scores from domain databases, and the list of domains we obtained do not natively map between databases, we created a variation of their algorithm by computing the product aggregation score for each set of domain annotations, and used logistic regression to compute a final score. We refer to this algorithm as the Domain Variant model. Finally, inspired by approaches that utilize a large set of features, we created a simple ensemble model using a random forest with features computed from best matching average aggregations for level 2 GO annotations in protein interactions, our 3 domains annotations in protein interactions, and Resnik semantic similarity as features [29,30]. See Supplementary Files S3 and S5 for full details of each algorithm.

### 2.4. Illogical/Random Feature Models to Serve as Control

Five different models were created that use either illogical or random features to demonstrate that the models may capture bias, rather than interaction-related properties, in datasets. By *illogical* feature, we mean that there is no logical reason to expect the feature to distinguish between interacting and non-interacting protein pairs. The expectation is that accurate PPI prediction methods should outperform these illogical/random models. See Supplementary Files S5 and S6 for full implementation details.

Count Bias simply counts how many more times the protein appears in positive training instances than negative training instances. A prediction score is simply the sum of these numbers for the two proteins in a data instance. Sequence Similarity Bias (Seq Sim Bias) is analogous to Count Bias, except that instead of counting each individual proteins’ appearance in the training data, it counts the positive and negative instances of up to the 5 most sequence similar proteins used in the training set, and performs a weighted average based on their similarity. A third feature, Sequence Similarity + Protein Bias (Seq Sim + Prot Bias), calculates the 5 most similar proteins, and their weights, from a combination of sequence similarity and the number of overlapping proteins shared by a given pair of Entrez IDs. This protein bias check is designed to ensure that non-sequence-based features (i.e., gene annotation features) are not getting an advantage when aggregating features from proteins that map to multiple genes. Sequence-based methods are not tested for this spurious feature. Finally, random features for each protein are represented by vectors of 500 random numbers, with pairs of proteins represented by a concatenation of these values. These features are then utilized by either a random forest or a simple neural network. The neural network architecture contains multiple shared layers before the protein pair’s values are concatenated and run through a final prediction layer.

### 2.5. Evaluation Metrics

For our evaluations, test sets with 50% positive data are compared on accuracy (Acc) and area under the receiver operating curve (AUC) measurements. These metrics allow us to compare our results with previous literature, which most commonly utilize accuracy to measure their model’s predictive capabilities. When data is imbalanced, as is common in the biological domain, typically having many more negatively labeled instances than positively labeled instances, precision recall curves (P-R) are recommended, as simple accuracy and AUC calculations may be heavily influenced by predicting the negatively labelled, non-rare class frequently [35,36]. Therefore, test sets with 10% or 0.3% positive data are compared on the precision at 3% recall (Prec) and average precision (Avg P), rather than relying on accuracy-based measures. 3% recall was chosen to determine whether the algorithms are capable of making good predictions on their top scoring pairs, a necessity to provide laboratories with good sets of protein pairs to test using experimental methods. See Supplementary File S7 for additional notes on implementing these metrics.

## 3. Results

**Figure 1:**
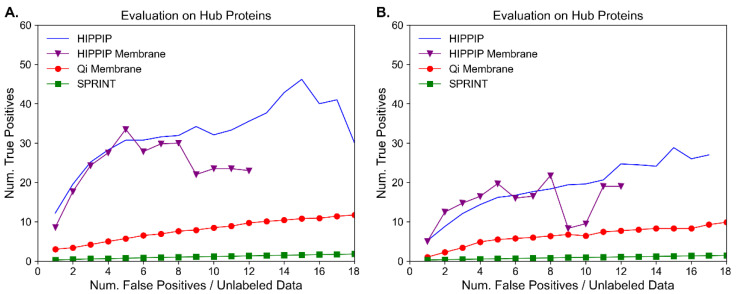
Evaluation of representative methods on hub proteins. Three methods (HiPPIP, that by Qi et al., and SPRINT) are evaluated on hub proteins. The figures show the cumulative number of true positives versus the number of false positives ^a^, computed individually for each protein and then averaged for each method for each point on *x*-axis. The hub proteins considered are: (**A**) same proteins as originally used in HiPPIP publication, and (**B**) hub proteins with more than 100 PPIs as per currently known PPIs from HPRD and BioGRID. The labeled data used in evaluation are from HPRD and BioGRID for (**A**) and (**B**). ^a^ Note that in this scenario of proteome-wide evaluation (i.e., where a hub protein is paired with every other protein in the human proteome), the unlabeled data may not all be false-positives, and may indeed me true positives that are currently unknown.

To illustrate the reasoning presented in the Introduction, we started by testing representative methods by evaluating their predictions on hub proteins against the known PPIs. First, HiPPIP is compared to Qi et al. as originally reported [37], but with recently updated data of known PPIs from HPRD and BioGRID. See Figure 1, where 1A is recomputed on the same hub proteins as in [37], and 1B is computed for hub proteins in current data with degree >100. In Figure 1B, a sequence-based predictor SPRINT [52], which in its original publication reported 79–89% AUC on 1:1 data composition, was generated from BioGRID; however, this high performance is not reflected at the proteome scale, and it is outperformed by the seminal work by Qi et al., which was published in 2009, and by HiPPIP.

We implemented various PPI prediction algorithms and tested most of them on their original datasets and evaluation metrics for verifying that the results are reproduced similarly. We created reusable datasets and evaluated the algorithms on these datasets, with commonly reported evaluation metrics as well as metrics more suitable for this domain, to benchmark their performance when predicting proteome-wide PPIs where interacting protein pairs are very rare. We suggested suitable evaluation metrics and are releasing the datasets and source code of re-implementations so that these together may lead to a rapid advancement of computational approaches for PPI prediction and their benchmarking.

### 3.1. Realistic Datasets for Benchmark Evaluations

We created benchmark training and test datasets at various proportions of positive class instances (1:1, 1:4, 1:9, and 1:332). In creating them, we employed two different approaches: the first approach selected pair-wise instances while ensuring that the pairs used in training and testing did not overlap (referred henceforth as Full data); the second approach held out proteins to be used exclusively in test data (referred henceforth as Held Out data). The size of each dataset is shown in Table 1.

### 3.2. Sequence-Based Predictors

We implemented 36 sequence-based PPI prediction algorithms with various different feature representations (Table 2) and evaluated them on the same datasets that were used in original publications (Table 3) to confirm that our implementations were comparable (Table 4). Excluding Tian et al.’s SVM algorithm, our implementations had <1% difference in accuracy on an average relative to those reported in those publications, with a correlation around 0.89. Large differences (>5%) were primarily observed when testing the smallest datasets (Martin Human and Martin H.Pylori). Overall, we obtained comparable results without extensive tuning or performing other significant hyperparameter optimization, showing that our implementations provide a good representation of previously produced works.

For each dataset used in previous literature, we compared our implementations of sequence-based predictors against the illogical/random feature control models. These comparisons are shown in Table 5. We can see that illogical/random feature models perform about as well as most previous implementations. In fact, the best result of each control model exceeds or falls within 4% accuracy of sequence-based predictors on all but two datasets. Our explanation is that sequence-based predictors likely capture information that some proteins are overrepresented in PPIs compared to random pairs (which is not an aspect that can be exploited in real-world interactome prediction). Thus, the datasets with much fewer proteins used in random pairs, such as Guo Multi Chen and both Pan Human datasets, are the easiest to obtain high accuracy on, as having several proteins exist in positive pairs without existing in negative pairs creates an easily exploitable bias when making predictions. These results strongly suggest that sequence-based prediction models inherently predict biases of individual proteins, or at least have been primarily evaluated on datasets where the biases are easy to exploit.

### 3.3. Benchmark Evaluation of Sequence-Based Methods

**Figure 2:**
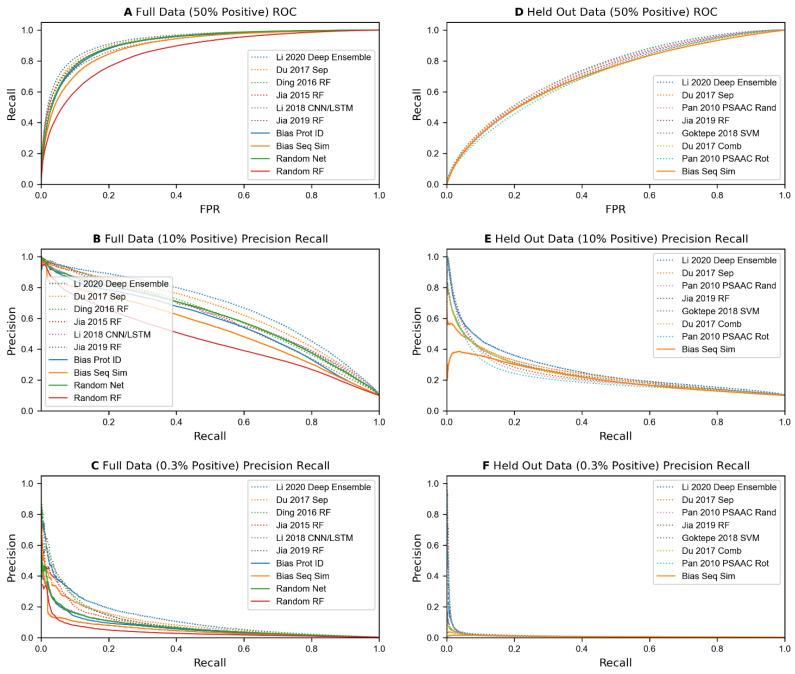
(**A**–**C**, **left**) Results when selecting from the full set of protein pairs for training and testing for a handful of the best algorithms (dotted lines) and the 4 illogical feature-based algorithms (solid lines). (**A**) ROC curve with 50% positive data. (**B**,**C**) Precision-Recall curves using 10% and 0.3% positive data in the test set. (**D**–**F, right**) Computations when performed on held out proteins instead of selecting from the full set of pairs. When holding out protein pairs, the algorithms exhibit a significant performance drop. Additionally, the algorithms and bias measurements score similarly across all 6 tests.

Each sequence-based predictor was trained on each of our 52 training datasets (26 based on Full data, and 26 based on Held Out data) and evaluated on the corresponding testing datasets (see Table 1). The results of these experiments are shown in Table 6 and Figure 2. Overall, most algorithms seem to be matched or outperformed by one or more of the control methods. Specifically, when scoring on datasets generated from the Full set of protein pairs with 1:1 positive class labels, using random numbers as features with a neural network model tied for 3rd in accuracy, and scored 5th in AUC in comparison to the dozens of sequence-based methods. In datasets where class distribution is skewed at 1:9, or more realistically at 1:332, this model placed 6th and 13th in average precision, outperforming over half of the sequence-based methods. Even when measuring precision at 3% recall on the skewed-class data, it outperforms half of the sequence-based predictors.

**Table 3:** Datasets reported in prior publications. The original publications that created these datasets are referenced in the first column. The next two columns report the species of the data and the publications that report PPI prediction accuracies on these data. The data sizes (number of PPIs are number of unique proteins) are reported after mapping protein names to UniProt and filtering to remove proteins shorter than 31 residues.

Dataset Creator	Species	Dataset-Referencing Paper	Positive Pairs	Random Pairs	Proteins in Positive Data	Proteins in Random Data
Du [56]	Yeast ^a^	Du [56]	17,257	48,594	4382	2521
Guo [9]	Yeast ^b^	Chen [66]	5594	5594	2217	2421
Guo [67]	Multi	Chen [66]	32,959	32,959	11,527	1399
Jia [57]	Yeast ^e,f^	Jia [57]	17,339	33,056	4436	3260
Liu [68]	Fruit Fly	Liu [68]	4156	4241	2463	4080
Martin [69]	H.Pylori	Jia [53]	1420	1458	1313	727
Martin [69]	Human	Pan [10]	937	938	828	740
Pan [10]	Human	Pan [10]	36,617	36,480	9473	2184
Pan [10]	Human	Pan [10]	3899	4262	2502	661
Guo [9]	Yeast ^b^	Tian [15]	5594	5594	2521	1194
Li [17]	Human ^c,d^	Li [17]	4096	4096	2805	1865
Richoux [22]	Human ^c^	Richoux [22]	39,672	64,388	6676	15,869

^a^ Only a random sample of random pairs are used to create a dataset of 50% positive data. ^b^ Different datasets based on Guo’s yeast data were found in literature. Unless specified otherwise, we use Tian’s dataset by default. ^c^ Data are provided in individual train and test sets, rather than used for cross validation. Test sets have fewer pairs than train sets. ^d^ Data from Alzheimer’s disease network. ^e^ Original dataset contained inter-species pairs. Pairs with non-yeast proteins were removed. ^f^ Jia’s yeast data is used in two different ways, split into a training/cross validation set of 50% positive data (Jia Yeast Cross) and a held out test set of 30% positive data (Jia Yeast Held), or for full cross validation (Jia Yeast Cross Full).

**Table 4:** Comparison of previous results to our implementations (increase or decrease of our implementations is listed in parenthesis, relative to the scores from the original publications).

Algorithm	Dataset	Accuracy	AUC
Guo 2008 AC SVM [9]	Guo Tian Yeast	87 (−2)	
Pan 2010 PSAAC SVM [10]	Martin Human	68 (−12)	
Pan 2010 PSAAC SVM [10]	Pan Small	91 (−18)	95 (−17)
Pan 2010 PSAAC Rot [10]	Pan Small	95 (+3)	97 (+2)
Pan 2010 PSAAC Rand [10]	Pan Small	96 (+2)	97 (+2)
Pan 2010 LDA Rot [10]	Pan Large	97 (+1)	99 (+0)
Pan 2010 LDA Rot [10]	Pan Small	96 (+2)	98 (+1)
Pan 2010 LDA Rand [10]	Pan Large	98 (+0)	99 (+0)
Pan 2010 LDA Rand [10]	Pan Small	96 (+2)	98 (+1)
Pan 2010 LDA SVM [10]	Martin Human	69 (−6)	
Pan 2010 LDA SVM [10]	Pan Large	95 (+2)	98 (+1)
Pan 2010 LDA SVM [10]	Pan Small	91 (+4)	95 (+3)
Pan 2010 AC SVM [10]	Martin Human	51 (+15)	
Pan 2010 AC SVM [10]	Pan Small	89 (+7)	94 (+4)
Pan 2010 AC Rot [10]	Pan Small	95 (+2)	96 (+3)
Pan 2010 AC Rand [10]	Pan Small	96 (+2)	97 (+2)
Zhou 2011 SVM [70]	Guo Tian Yeast	89 (+2)	95 (+1)
Zhao 2012 SVM [11]	Liu Fruit Fly	81 (−4)	
Zhao 2012 SVM [11]	Martin H Pylori	89 (−3)	
Jia 2015 RF [57]	Jia Yeast Held	87 (−5)	
Jia 2015 RF [57]	Jia Yeast Cross	84 (−6)	
Jia 2015 RF [57]	Martin H Pylori	91 (−3)	
You 2015 RF [61]	Guo Tian Yeast	95 (−1)	
You 2015 RF [61]	Martin H Pylori	88 (−2)	
Ding 2016 RF [63]	Guo Tian Yeast	95 (−1)	
Ding 2016 RF [63]	Martin H Pylori	88 (+2)	
Ding 2016 RF [63]	Pan Small	98 (+0)	
Du 2017 Sep [56]	Du Yeast	93 (+0)	97 (−0)
Du 2017 Sep [56]	Guo Tian Yeast	94 (+1)	
Du 2017 Sep [56]	Martin H Pylori	86 (+2)	
Du 2017 Sep [56]	Pan Small	98 (+1)	
Du 2017 Comb [56]	Du Yeast	90 (+1)	96 (+0)
Sun 2017 CT Auto [12]	Pan Large	95 (−1)	
Sun 2017 AC Auto [12]	Pan Large	97 (−0)	
Wang 2017 Rot [19]	Guo Tian Yeast	90 (−0)	
Wang 2017 Rot [19]	Martin H Pylori	88 (−12)	
Göktepe 2018 SVM [13]	Martin Human	74 (−6)	83 (−11)
Göktepe 2018 SVM [13]	Martin H Pylori	89 (−5)	94 (−3)
Göktepe 2018 SVM [13]	Pan Small	94 (+4)	93 (+6)
Gonzalez-Lopez 2018 [21]	Du Yeast	93 (−1)	97 (−0)
Gonzalez-Lopez 2018 [21]	Guo Tian Yeast	95 (−1)	98 (−0)
Gonzalez-Lopez 2018 [21]	Martin H Pylori	85 (+1)	92 (−0)
Gonzalez-Lopez 2018 [21]	Pan Small	98 (+1)	100 (−0)
Hashemifar 2018 CNN [20]	Guo Tian Yeast	95 (+0)	
Li 2018 CNN/LSTM [23]	Pan Large	99 (−0)	
Chen 2019 LGBM [14]	Guo Tian Yeast	95 (−0)	
Chen 2019 LGBM [14]	Martin H Pylori	89 (−1)	
Chen 2019 RNN [66]	Guo Chen Yeast	97 (−1)	
Jia 2019 RF [53]	Jia Yeast C. Full	88 (−4)	
Jia 2019 RF [53]	Martin H Pylori	93 (−4)	
Richoux 2019 LSTM [22]	Richoux Strict	78 (−1)	
Richoux 2019 Full [22]	Richoux Strict	76 (−1)	
Tian 2019 SVM [15] ^a^	Guo Tian Yeast	96 (−12)	
Tian 2019 SVM [15] ^a^	Martin H Pylori	96 (−17)	
Yao 2019 Net [71]	Guo Tian Yeast	95 (+1)	
Yao 2019 Net [71]	Pan Small	99 (+0)	
Zhang 2019 Deep [16]	Du Yeast	95 (−4)	97 (−2)
Li 2020 Deep [17]	Li AD	95 (+3)	95 (+5)
Czibula 2021 Auto SS [18]	Guo Chen Multi	97 (+0)	97 (+2)
Czibula 2021 Auto SS [18]	Pan Large	98 (−1)	98 (+1)
Czibula 2021 Auto SJ [18]	Guo Chen Multi	97 (−0)	97 (+2)
Czibula 2021 Auto SJ [18]	Pan Large	98 (−1)	98 (+1)
Czibula 2021 Auto JJ [18]	Guo Chen Multi	98 (−1)	98 (+1)
Czibula 2021 Auto JJ [18]	Pan Large	98 (−1)	96 (+3)

^a^ Results of Tian are expected to be different due to differences in data preprocessing, see S6 for details.

**Table 5:** Comparisons of the range of values per dataset in previous literature and our implementations, versus 4 algorithms that rely on simple counting or random numbers. Only 1 dataset was successfully predicted by an algorithm at a rate of over 10% above using random numbers, while most algorithms struggle to predict any better than counting or using random numbers per protein. Even the best results from each of the three datasets used in 10 or more previous implementations only exceed predictions using random numbers by less than 3%, with several algorithms falling below the bias-based predictions.

Dataset Referred by Table 1 Column 1, Column 2, Column 3	Count Bias	Seq Sim Bias	Rand Net	Rand RF	Number of Implementations	Results Reported in Publications	Results of Our Implementations	Max Improvement over Bias Methods
Du Yeast	87.7	87.5	92.5	88.5	4	90–95.3	90.5–92.6	2.8%
Guo Yeast Chen	81.5	81.4	84	74.4	1	97.1	96.3	13.1%
Guo Yeast Tian	87	85.9	94.3	94	10	87.3–95.1	84.5–95.5	1.2%
Guo Multi Chen	93.5	93.1	98.7	96.4	3	96.9–98.2	96.6–97.3	−0.5%
Jia Yeast Cross	78.7	78.4	75.2	76.5	1	84.4	77.9	5.7%
Jia Yeast Held	82.9	82.5	81.3	80.7	1	86.5	82.0	3.6%
Jia Yeast C. Full	83.8	83.1	85.4	81.1	1	88	84.3	2.6%
Li AD	96.7	79.1	76.2	97.3	1	94.7	97.7	0.4%
Liu Fruit Fly	84.1	95.7	96.6	84.2	1	80.9	76.8	−15.7%
Martin Human	61.2	83.1	81.1	62.2	4	51–73.8	55.7–67.8	−9.3%
Martin H Pylori	83.6	61	59.2	89.6	10	85.2–93	75.9–89.9	3.4%
Pan Human Large	96.3	83.1	82.2	97.8	9	94.5–99	94.3–98.9	1.2%
Pan Human Small	94.5	93.1	98.8	98.6	14	89.3–98.7	72.5–99.4	0.6%
Richoux Strict	79.6	94.4	96.9	79.5	2	76.3–78.3	74.7–76.8	−18.6%

**Table 6:** Accuracies, Area Under the Receiver Operating Curve, Precision, and Average Precisions across all sequence based and illogical feature-based models and all datasets.

Algorithm	50% Pos Full		10% Pos Full		0.3% Pos Full		50% Pos Held		10% Pos Held		0.3% Pos Held	
	Acc	AUC	Prec	Avg P	Prec	Avg P	Acc	AUC	Prec	Avg P	Prec	Avg P
Control Methods												
Count Bias	84.2	91.5	91.9	56.4	28.0	6.5	50.0	50.0	10.0	10.0	0.3	0.3
Seq Sim Bias	82.3	90.0	84.5	53.0	14.8	5.2	65.6	70.7	41.8	21.6	2.1	0.9
Random Vec NNet	84.7	92.0	92.5	60.4	29.1	7.4	51.0	50.2	11.1	10.0	0.4	0.3
Random Vec RF	78.1	85.9	87.3	45.9	17.9	3.8	50.9	50.5	10.5	10.1	0.3	0.3
Sequence-Based Predictors												
Guo 2008 AC SVM [9]	74.1	81.5	83.3	40.4	16.1	3.4	61.4	65.4	44.2	17.3	2.4	0.7
Pan 2010 PSAAC SVM [10]	64.2	68.4	46.7	21.3	2.7	1.4	63.2	67.1	43.5	19.2	2.2	1.1
Pan 2010 PSAAC Rot [10]	82.9	90.6	96.1	57.5	44.5	8.5	64.4	70.2	62.1	20.8	4.9	1.4
Pan 2010 PSAAC Rand [10]	83.7	91.4	96.8	59.9	46.9	9.3	66.4	72.5	65.7	22.6	5.4	1.5
Pan 2010 LDA Rot [10]	82.7	90.4	93.7	54.7	33.7	6.8	59.7	63.9	38.0	16.6	2.3	0.9
Pan 2010 LDA Rand [10]	83.5	91.1	94.2	57	35.9	7.4	61.3	65.9	44.6	17.9	3.1	1.0
Pan 2010 LDA SVM [10]	77.8	85.3	88.4	45.9	19.2	4.2	58.8	61.9	27.4	14.9	1.3	0.5
Pan 2010 AC SVM [10]	80.2	87.2	85.5	47.9	13.6	3.8	59.9	64.5	41.8	18.2	2.0	0.7
Pan 2010 AC Rot [10]	83.2	91.0	94.3	55.2	37.7	6.8	57.8	61.3	30.7	14.5	1.3	0.7
Pan 2010 AC Rand [10]	83.9	91.7	94.1	57.0	39.0	7.4	59.8	64.3	37.0	15.4	2.1	0.8
Zhou 2011 SVM [70]	80.4	88.2	89.0	51.7	23.6	5.4	60.6	64.5	33.6	17.1	1.5	0.6
Zhao 2012 SVM [11]	77.9	83.5	86.6	39.6	14.9	2.9	64.4	68.5	35.0	19.1	1.7	0.7
Jia 2015 RF [57]	84.6	92.2	95.9	60.7	41.8	8.7	65.1	70.4	51.1	19.5	3.3	1.2
You 2015 RF [61]	83.1	90.8	95.8	56.5	43.9	7.7	61.2	65.9	42.9	17.4	2.5	1.0
Ding 2016 RF [63]	84.7	92.3	96.9	61.1	49.8	10.0	64.1	70.0	58.3	18.3	4.6	1.2
Du 2017 Sep [56]	85.5	92.8	94.9	64.5	39.5	9.8	67.0	73.3	56.3	24.9	3.9	1.2
Du 2017 Comb [56]	83.2	90.6	94.7	59.0	33.8	7.9	65.1	70.7	58.5	23.5	4.1	1.1
Sun 2017 CT Auto [12]	74.4	82.0	61.7	37.8	4.3	2.0	58.8	62.2	26.9	14.4	1.0	0.5
Sun 2017 AC Auto [12]	77.3	84.4	74.1	42.5	8.8	2.9	58.1	60.5	22.4	14.5	0.8	0.5
Wang 2017 Rot [19]	70.2	73.5	33.0	21.8	1.3	0.8	56.2	57.1	16.1	11.8	0.5	0.4
Göktepe 2018 SVM [13]	82.5	90.2	93.6	57.0	29.2	7.1	65.6	71.2	59.4	24.0	4.6	1.2
Gonzalez-Lopez 2018 [21]	83	90.5	89.9	55.6	23.9	5.9	54.1	55.4	17.8	11.9	0.7	0.4
Hashemifar 2018 CNN [20]	82.2	89.3	84.8	48.9	15.0	3.9	61.4	65.5	30.8	16.8	1.4	0.6
Li 2018 CNN/LSTM [23]	84.3	91.8	93.6	60.9	28.3	7.3	56.0	58.5	24.7	13.6	1.1	0.5
Chen 2019 LGBM [14]	81.9	89.7	96.0	57.6	45.2	8.4	62.7	67.7	43.6	19.8	2.4	1.0
Chen 2019 RNN [66]	83.9	90.4	75.4	54.7	7.7	4.0	59.8	63.2	27.1	15.7	1.2	0.5
Jia 2019 RF [53]	83.2	91.0	97.1	59.5	52.7	10.0	66.0	72.0	68.2	23.2	5.6	1.6
Richoux 2019 LSTM [22]	80.0	87.0	91.6	52.8	25.2	5.8	54.3	55.5	15.6	11.9	0.6	0.4
Richoux 2019 Full [22]	82.8	90.4	91.7	57.9	25.7	6.5	55.2	56.8	15.1	12.2	0.6	0.4
Tian 2019 SVM [15]	76.0	83.6	86.4	44.3	18.8	4.1	65.3	70.8	57.7	22.0	4.3	1.0
Yao 2019 Net [71]	83.5	90.7	89.3	55.9	20.1	5.6	57.7	60.7	27.1	14.7	1.2	0.5
Zhang 2019 Deep [16]	81.4	88.1	74.8	51.0	8.0	3.7	59.5	61.7	38.2	17.1	1.9	0.6
Li 2020 Deep [17]	86.4	93.4	96.6	67.7	50.7	12.4	67.3	73.8	65.9	26.8	6.1	1.7
Czibula 2021 Auto SS [18]	76.5	84.7	66.8	36.5	5.7	2.1	53.1	54.0	11.3	11.3	0.7	0.4
Czibula 2021 Auto SJ [18]	66.6	74.8	66.6	33.3	5.9	1.8	53.0	54.4	18.2	11.6	0.8	0.4
Czibula 2021 Auto JJ [18]	74.7	82.6	67.9	35.8	5.8	2.1	55.7	57.2	40	12.9	1.7	0.5

When testing on protein pairs containing Held Out proteins for test data, the accuracies and precisions of all published algorithms are much lower, with the best prediction accuracy on 1:1 data falling below 70%, and the precision at 3% recall and average precision on the 1:332 data falling below 10% for all models. Biases that exploit the number of positive and negative instances in the training data that each protein from the test dataset appear in, such as simple counts and random numbers, are eliminated when using our Held Out protein data generation method. This is shown by their prediction accuracies and precisions reducing down to being nearly equal to the percentage of positive data in the test set, implying random ordering. However, predicting on individual proteins based on sequence similarity still places in the top half of all comparisons, and in the top 10 in accuracy and AUC on 50% positive test data.

### 3.4. De-Biasing Annotation-Based Predictors

Computations based on gene annotation features (non-sequence-based) can induce biases when dealing with missing data. If the missing data is much more prominent in the positive or negative class (which it can be for PPI prediction datasets [72]), algorithms can learn to make predictions based on number of missing values as a spurious feature. In Table 7, we show the amount of missing data for each dataset. While negative pairs have more missing data, most pairs contain at least one of the three GO features, and some Pfam and InterPro features. Given that more than half of the negative data contain information from all 3 GO ontologies, more than 40% of them contain domain data from Prosite, the feature that is most often missing, combined with the heavy skew of negative data in the imbalanced datasets, we do not believe that, from these features, missing data alone would provide a significant advantage to predicting PPIs.

Additionally, many of the published algorithms based on protein domains, and to a lesser extent on GO annotations, utilized information related to interactions either directly, by computing the probability that a pair of annotations belongs to known interactions, or indirectly by utilizing a domain database that bases its scores on the probability of a pair of domains belonging to protein interactions. When utilizing these data without filtering out interacting pairs in the test data, the results could be biased such that rare pairs of domains or GO annotations that occur in an interaction in the test dataset are scored highly solely because of that interaction’s usage when generating the protein pair’s features. The performance of our two algorithms using features that rely on interactions is shown in Table 8 when allowing the features to be created using all, non-testing, and non-held out proteins exclusively. Overall, there is a significant drop in accuracy when removing test pairs from the feature creation process, with a more moderate drop when holding out entire proteins instead of just the pairs in the test dataset. As test data should not influence feature creation and training, we believe this shows a significant bias which must be accounted for when calculating features. However, compared to sequence-based predictors, the drop found when changing from holding out test data to holding out entire proteins is much more moderate, suggesting less of a biasing issue when not holding out entire proteins. While the difference is smaller, for the purpose of fair comparisons, we utilized features calculated with proteins being held-out when comparing to sequence-based methods on the Held Out datasets.

### 3.5. Benchmark Evaluation of Annotation-Based Methods

**Figure 3:**
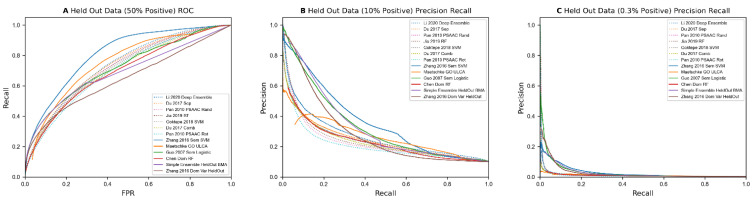
(**A**). ROC curve for results on held out proteins on 50% positive data, comparing sequence (dotted lines) an annoation-based methods (solid lines). (**B**,**C**). Precision Recall Curves for held out proteins on 10% and 0.3% positive data, comparing sequence and non-sequence-based methods. Non-sequence-based methods (solid lines) perform better at lower recall levels when positive data is more rare than random pairs.

We tested our implementations of annotation-based methods on the benchmark datasets. These results, along with predictions from illogical/random feature models and some of the best sequence-based predictors, are shown in Table 9. Overall, the results from these methods are worse than control models in several categories when not holding out any data. However, unlike control and sequence-based methods, the four methods that use small feature vectors not relying heavily on individual protein data (i.e., excluding ‘Chen 2005 Dom RF’ and ‘Maetschke 2012 ULCA’) drop much less when running on held out data, with some even improving their precision at 3% recall. Unlike the sequence-based methods, these 4 methods manage to obtain over 85% precision at 3% recall on 1:9 positive data, and 15–25% precision on 1:332 data. The latter result represents a 50x–80x performance improvement over random data, over a 7x improvement over predicting based on similar sequences, and a 2x–4x improvement over the best sequence-based methods at low recall levels. While methods using aggregations showed the ability to make good predictions at low recall levels, Chen et al.’s method using the pairwise sum of binary data per protein and Maetschke’s method using a union of GO annotations between two proteins up to and including their lowest common ancestor both struggled like sequence-based methods. This may be a reflection of the large number of features used by these models, as well as how the feature vectors utilized mostly reflect each individual protein’s features or a simple calculation between individual protein’s features. A plot of the best annotation-based predictors versus the best sequence-based predictors can be found in Figure 3. In both precision recall curves, there is a clear gap in precision between the 4 best annotation-based predictors and sequence-based predictors at low recall levels.

## 4. Discussion

We implemented different PPI prediction algorithms and evaluated them on benchmark datasets containing different class distributions. Our results show that most published methods perform much lower than originally reported when evaluation data are created with realistic proportions of positive and negative classes. We also showed that many of these methods may be capturing spurious, illogical features that represent the frequency of specific proteins in the data rather than meaningful information about PPIs themselves; such methods will not translate to a real-world application of proteome-wide discovery of PPIs where every protein will be tested against every other protein in the proteome.

In prior publications, most sequence-based predictors were evaluated on datasets with 50% positively labeled instances, with randomly selected protein-pairs serving as negative class data. In those datasets, PPIs (i.e., the positive class data) are drawn from a scale-free network where some nodes are hubs, and therefore appear in many protein pairs, whereas randomly paired negative class data are drawn from nearly complete-graph data. Thus, the usage rates of different proteins, particularly hub proteins, in the positively and negatively labeled instances are dramatically different, creating an easily exploitable bias. Some of this can be inferred from Table 3, which shows far more unique proteins in positive class data than in negative class data in many datasets. (If say 100 pairs are drawn from scale-free distribution and there is one hub with 25 PPIs, it contains 26 unique proteins; whereas, if that hub did not exist and it was drawn from a complete graph, the number of unique proteins from those 25 PPIs would be between 26 and 50) When evaluating these datasets, algorithms may assign class labels based on protein frequency rather than true characteristics of an interacting protein pair, and falsely shows higher performance in evaluations; the class label is assigned independently of the second protein in the pair but learns a likely invalid premise for real world interactome prediction. Our experiments showed that both on datasets from original publications and on our newly created Full datasets, control models with illogical features that simply capture protein membership can perform on par with most of the models from the literature. When analyzing the results of test datasets with proteins not utilized in the training set (Held Out), we find that most algorithms’ accuracies drop significantly. Accuracies on our 1:1 test data dropped from their original results of 75–85% accuracy down to 55–67% accuracy. On more relevant metrics, namely precision at 3% recall on 1:332 positive class data and holding out test proteins gave a mere 6.1% for the best sequence-based algorithm. Thus, when both the data ratio and evaluation metric are suitably chosen, the true ability is revealed to be impractically low.

Some of the previous methods filtered out protein sequences that have a high sequence similarity, which we have not implemented; however, we have mapped our data to gene-level information, and so multiple isoforms are not included in our test data, minimizing the number of highly sequence similar proteins in our data. It is likely that if we removed proteins with similar sequences from our datasets, the results when predicting on held out proteins would be lower, as exploiting sequence similarity provides similar results to sequence-based methods. This may be analyzed in future work.

When training the methods from literature that were originally designed for 50% positive training and test datasets, we did not make adjustments to the designs or implementations to adjust for the different ratios of data. Some methods, such as class weighting, are commonly recommended when training models on imbalanced data. However, to keep the models as similar to those found in previous literature as possible, we decided not to implement adjustments per positive data ratio. We note that we did test all models on 50% positive test data and found the results to be similar to what could be obtained by prioritizing proteins by their number of known interactions. Therefore, we believe it is unlikely that using concepts such as class-weighting would drastically increase the precision of these methods.

When using annotation features, we found that feature representation has a significant impact on the results of the model. Most methods we tested were unable to outperform control models made from illogical features when generating data from all pairs of proteins. However, using Held Out protein data showed moderate precision at 3% recall even on heavily imbalanced class data, showing their predictive capabilities do not depend on exploitable, individual protein-based biases in the underlying data.

Models that use features computed from pairs of protein domains and GO annotations that appear in other interactions performed well at predicting interactions; these are well recognized to be meaningful features in predicting interactions (e.g., that two protein domains that are known to interact are highly likely to conserve that interaction/function when those protein domains appear in other proteins). Thus, using the knowledge of interacting protein domains or compatible GO annotations (specific ligand and receptor annotation in the two proteins), and along with other protein features to learn an effective classification machine learning model which helps shortlist protein-pairs for experimental validation. We also note that the two methods using only 3 features, i.e., ‘Domain Variant’ and ‘Guo 2006 Sem’, performed as well or better than other non-sequence methods based on 10–20 features. Surprisingly, our ensemble method, which contained Resnik semantic similarity, GO annotation frequency, and all 3 domain features performed worse than the methods using only 3 domain or Resnik semantic similarity features. This was most likely due to the different aggregation methods, with Domain Variant and Guo 2006 Sem using product and max aggregations respectively, while our ensemble used best matching average aggregation. This could imply that using max or product aggregation is better for predicting protein interactions, or this could suggest a bias where protein interactions are primarily known for genes with a high number of annotations. If the latter is true, product, sum, and maximum aggregations could exploit this bias, as all three functions monotonically increase as more data are provided. We leave analyzing this to future work.

As for methods where the features used by the models were highly similar to features produced for each individual protein, such as Chen 2005 Dom RF and Maetschke 2012 ULCA, we found that their performance mirrored the performances of sequence-based methods. This implies that using sequences alone is not the problematic part for sequence-based methods, but rather, any methodology that relies on producing unique feature sets per protein and using simple combinations of these features to create data for machine learning methods seem to mostly make predictions based on underlying biases in generated PPI datasets. Only when creating a small number of more complex features using pairs of proteins, instead of individual proteins, do models see a significant improvement beyond bias when positive interaction data are used as the rare class.

**Figure 4:**
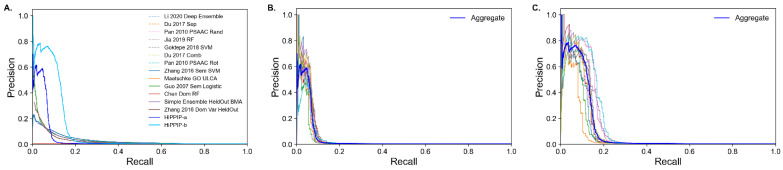
Evaluation of HiPPIP. (**A**) Comparison of HiPPIP with selected sequence-based and annotation-based methods that performed the best. The two lines HiPPIP-a and HiPPIP-b correspond to whether the known PPIs in the test data were drawn from recent version of BioGRID (May 2021, same as used in this paper) or from an earlier version of BioGRID (January 2017). Both lines in (**A**) show an average performance across 10 datasets, whereas performance on individual datasets in each set are shown in (**B**) and (**C**), respectively. For all methods, including sequence-based and annotation-based methods, pairs that appear in the HPRD database are also treated as true-positives in this figure; there is no discernable difference between the results of the sequence-based and annotation-based algorithms in Figure 3C and here, despite the inclusion of HPRD PPIs as positive labels. It is estimated that HiPPIP-a and HiPPIP-b test datasets may have about 24% and 14% overlap for positive instances respectively, because the specific pairs used in training HiPPIP are unavailable.

In conclusion, we compare P-R performance of HiPPIP with selected sequence-based and annotation-based methods (Figure 4A) that performed the best and find that HiPPIP outperforms all of them significantly. In this evaluation, for all predictors, known PPIs from HPRD dataset are also included in assessing whether a prediction is a true positive. HiPPIP was evaluated on two sets of test data, each set containing 10 datasets: in one set, the data are created in the same fashion as Full data (‘HiPPIP-a’ in Figure 4A) and in the other, the positive instances for test data are taken from BioGRID published January 2017 (‘HiPPIP-b’ in Figure 4A); Figure 4 shows aggregated results across the datasets in 4A and individual datasets in Figure 4B,C for the two sets. The aforementioned caveat is: the specific pairs used in training HiPPIP are unavailable; therefore, there may be some overlap with training data; based on the number of known PPIs in BioGRID and considering that 20,000 PPIs were used in training HiPPIP, it is estimated that HiPPIP-a and HiPPIP-b test datasets may have 24% and 14% overlap for positive instances respectively, whereas overlap for negative data are negligible.

## 5. Conclusions

In this analysis, we compared protein interaction predictions from a variety of different algorithms in the literature, including methods based on sequences, Gene Ontology, and domain features. Overall, we found significant biases in several previous studies. When removing these biases, our results showed that pairwise features, such as semantic similarity and pairs of domains existing in known protein interactions, predicted interactions in highly imbalanced data at low recall levels that outperformed other competing methods, such as those using sequence-based features. When using methods involving feature sets per protein, as is done with most sequence-based predictions, we found that most algorithms fail to predict interactions significantly better than simple illogical features based on individual proteins.

In these implementations, we did not make any significant adjustments or tuning of the algorithm hyperparameters in order to compare these results with original reported results. We believe that by releasing the source code and the datasets, the algorithms will continue to be improved by the scientific community by devising better features and algorithms, or even by tuning the algorithms to handle the underlying skewed distribution.

## Figures and Tables

**Table 1:** Sizes of different datasets created for testing. Pair sizes vary for the held-out data due to the number of pairs drawn from a single bin (such as 4, 4) being half the size of drawing pairs from 2 bins (such as 3, 4). Held out test sets for 1:1 (50%) and 1:9 (10%) positive pairs use all positive data per held out bin.

Pos Ratio	Usage	Instance Sampling	Number of Datasets	Positive Pairs	Negative Pairs	Proteins in Positive Data	Proteins in Negative Data
1:1 (50%)	Both	Full	1 (5-fold CV)	62,500	62,500	12,895	19,082
1:4 (20%)	Train	Full	5	20,000	80,000	9250–9345	19,105–19,110
1:9 (10%)	Test	Full	5	10,000	90,000	6868–6987	19,109–19,111
1:332 (0.3%)	Test	Full	5	1500	498,500	2099–2170	19,112
1:1	Train	Held Out	21	50,000	50,000	8849–10,762	12,734–15,899
1:1	Test	Held Out	21	3170–7129	3170–7129	1530–3233	2770–5707
1:4	Train	Held Out	21	20,000	80,000	6978–8297	12,749–15,927
1:9	Test	Held Out	21	3170–7129	28,530–64,161	1530–3233	3185–6372
1:332	Test	Held Out	21	300–600	99,700–199,400	398–846	3185–6372

**Table 2:** Different preprocessing algorithms used on amino acid sequences prior to model training.

Amino Acid Composition (AAC)	Auto Covariance (AC) [9]	Chaos Game Representation [53]	Conjoint Triad (CT) [54]	Composition-Transition-Distribution (CTD) [55]
Dipeptide Composition [56]	Discrete Wavelet Transform Physicochemical [57]	Encoding Based on Grouped Weight (EBGW) [58]	Geary Autocorrelation [59]	Local Descriptor (LD) [60]
Multi-scale Local Descriptor [61]	Multi-scale Continuous and Discontinuous [62]	Multivariate Mutual Information [63]	Moran Autocorrelation [59]	Normalized Moreau-Broto Autocorrelation [59]
Numeric/One Hot encoding	Pseudo Amino Acid Composition [64]	PSSM ([46])	PSSM(DPC)/PSSM(Bi-gram) [13]	PSSM Discrete Cosine Transform [19]
Quasi Sequence Order Descriptor [65]	Sequence Order [11]	Skip Gram [45]	Weighted Skip-Sequential Conjoint Triad [13]	

**Table 7:** Percentages of missing data in the positive and negative our benchmark datasets. Overall, 1–15% more negative data is missing from most datasets in each category. However, most pairs appear to have at least some data, as shown by the GO Any and InterPro columns, each showing at least 94–97% of all protein pairs have some data from GO and domains.

Dataset	Pos%	Data Type	Class	GO CC	GO BP	GO MF	GO Any	Pfam	Prosite	InterPro
Full	50%	Train	Positive	7.3	13.3	2.9	0.7	8.2	47.8	1.2
Full	50%	Train	Negative	13.6	22.2	17.9	5.9	11.9	57.7	2.3
Full	50%	Test	Positive	7.3	13.3	2.9	0.7	8.2	47.8	1.2
Full	50%	Test	Negative	13.6	22.2	17.9	5.9	11.9	57.7	2.3
Full	20%	Train	Positive	7.2	13.2	2.8	0.7	8.2	47.6	1.2
Full	20%	Train	Negative	13.8	22.2	17.9	6.0	12.0	57.8	2.4
Full	10%	Test	Positive	7.2	13.2	2.9	0.7	8.2	48.0	1.2
Full	10%	Test	Negative	13.7	22.0	17.9	6.0	12.1	57.8	2.4
Full	0.3%	Test	Positive	7.3	13.3	2.7	0.7	7.9	48.6	1.1
Full	0.3%	Test	Negative	13.7	22.1	17.9	6.0	12.0	57.8	2.4
Held Out	50%	Train	Positive	7.2	13.3	2.8	0.7	8.2	47.9	1.2
Held Out	50%	Train	Negative	13.7	22.0	17.8	6.0	12.0	57.8	2.4
Held Out	50%	Test	Positive	7.2	13.2	2.8	0.7	8.1	47.9	1.2
Held Out	50%	Test	Negative	13.9	22.1	17.9	6.1	12.0	57.6	2.3
Held Out	20%	Train	Positive	7.2	13.3	2.8	0.7	8.1	48.0	1.2
Held Out	20%	Train	Negative	13.7	22.0	17.9	6.0	12.0	57.8	2.4
Held Out	10%	Test	Positive	7.2	13.2	2.8	0.7	8.1	47.9	1.2
Held Out	10%	Test	Negative	13.6	22.0	17.9	5.9	12.0	57.8	2.4
Held Out	0.3%	Test	Positive	6.5	12.9	2.9	0.7	7.7	47.3	1.2
Held Out	0.3%	Test	Negative	13.7	22.0	17.9	6.0	12.0	57.7	2.4

**Table 8:** Accuracy of a domain feature predictor (Zhang 2016 Domain Variant) and ensemble (Simple Ensemble) predictor using all interactions, non-test interactions, and non-held out protein pair interactions when computing features. Held out protein pairs are not valid for datasets created by selecting random pairs. The accuracy of these predictors drops significantly when removing the test interactions from the feature creation process.

Algorithm	50% Pos Rand		10% Pos Rand		0.3% Pos Rand		50% Pos Held		10% Pos Held		0.3% Pos Held	
	Acc	AUC	Prec	Avg P	Prec	Avg P	Acc	AUC	Prec	Avg P	Prec	Avg P
Dom Var All	92.0	97.3	99.0	88.4	72.9	42.4	92.1	97.3	99.1	88.3	76.2	43.7
Dom Var NonTest	74.2	77.4	96.7	51.5	41.3	9.0	73.8	76.9	96.4	49.9	46.4	9.6
Dom Var HeldOut							63.3	64.2	91.8	28.7	25.6	2.8
Ensemble All	94.3	98.3	97.5	90.9	51.3	38.3	93.8	98.0	97.5	90.1	52.2	38.0
Ensemble NonTest	76.4	82.2	92.2	54.1	25.4	8.8	76.0	81.4	92.1	51.9	23.9	8.0
Ensemble HeldOut							64.7	67.2	88.4	27.9	16.4	2.3

**Table 9:** Results of non-sequence-based predictors versus control and annotation-based predictors.

Algorithm	50% Pos Full		10% Pos Full		0.3% Pos Full		50% Pos Held		10% Pos Held		0.3% Pos Held	
	Acc	AUC	Prec	Avg P	Prec	Avg P	Acc	AUC	Prec	Avg P	Prec	Avg P
Annotation-Based Methods												
Chen 2005 Dom RF [26]	75.9	82.7	77.1	41.8	8.8	2.8	65.1	69.6	58.8	23.1	4.3	1.0
Gou 2006 Sem [24]	66.3	72.1	90.1	33.7	15.7	2.9	66.3	72.2	89.5	33.1	22.1	3.3
Maetschke 2012 ULCA [28]	71.0	77.4	46.2	28.6	2.3	1.2	69.5	75.2	42.0	25.5	2.0	1.0
Dom Variant	74.2	77.4	96.7	51.5	41.3	9.0	63.3	64.2	91.8	28.7	25.6	2.8
Zhang 2016 Sem [25]	73.5	80.4	86.0	35.7	13.5	2.7	73.0	79.5	85.4	33.4	19.7	2.9
Simple Ensemble	76.4	82.2	92.2	54.1	25.4	8.8	64.7	67.2	88.4	27.9	16.4	2.3
Control Methods												
Count Bias	84.2	91.5	91.9	56.4	28.0	6.5	50.0	50.0	10.0	10.0	0.3	0.3
Seq Sim Bias	82.3	90.0	84.5	53.0	14.8	5.2	65.6	70.7	41.8	21.6	2.1	0.9
Seq Sim Bias + Protein Bias	82.3	89.9	84.5	52.9	14.8	5.2	65.6	70.8	41.6	21.6	2.1	0.9
Rand Net	84.7	92.0	92.5	60.4	29.1	7.4	51.0	50.2	11.1	10.0	0.4	0.3
Rand RF	78.1	85.9	87.3	45.9	17.9	3.8	50.9	50.5	10.5	10.1	0.3	0.3
Selected Best-Performing Sequence-Based Methods												
Pan 2010 PSAAC Rand [10]	83.7	91.4	96.8	59.9	46.9	9.3	66.4	72.5	65.7	22.6	5.4	1.5
Jia 2015 RF [57]	84.6	92.2	95.9	60.7	41.8	8.7	65.1	70.4	51.1	19.5	3.3	1.2
Ding 2016 RF [63]	84.7	92.3	96.9	61.1	49.8	10.0	64.1	70.0	58.3	18.3	4.6	1.2
Du 2017 Sep [56]	85.5	92.8	94.9	64.5	39.5	9.8	67.0	73.3	56.3	24.9	3.9	1.2
Göktepe 2018 SVM [13]	82.5	90.2	93.6	57.0	29.2	7.1	65.6	71.2	59.4	24.0	4.6	1.2
Li 2018 CNN/LSTM [23]	84.3	91.8	93.6	60.9	28.3	7.3	56.0	58.5	24.7	13.6	1.1	0.5
Jia 2019 RF [53]	83.2	91.0	97.1	59.5	52.7	10.0	66.0	72.0	68.2	23.2	5.6	1.6
Li 2020 Deep [17]	86.4	93.4	96.6	67.7	50.7	12.4	67.3	73.8	65.9	26.8	6.1	1.7

## Data Availability

The benchmark datasets and source code of our implementations of various published algorithms can be downloaded from https://github.com/bmd2007/benchmark_eval.

## Supplementary Materials

The following are available online. S1: Details of downloading and processing positive protein interactions. S2: Implementation details of sequence-based features. S3: Implementation details of annotation-based features. S4: Details of classification models and hyperparameter processing. S5: List and brief descriptions of published algorithms that are re-implemented here. S6: Implementation details of control models with illogical and random features. S7: Evaluation Metric Notes.

## References

[1] Tang Z., Takahashi Y. (2018). Analysis of Protein–Protein Interaction by Co-IP in Human Cells. Two-Hybrid Systems.

[2] Johnson K.L., Qi Z., Yan Z., Wen X., Nguyen T.C., Zaleta-Rivera K., Chen C.J., Fan X., Sriram K., Wan X. (2021). Revealing protein-protein interactions at the transcriptome scale by sequencing. Mol. Cell.

[3] Huang H., Jedynak B.M., Bader J.S. (2007). Where have all the interactions gone? Estimating the coverage of two-hybrid protein interaction maps. PLoS Comput. Biol..

[4] Luck K., Kim D.-K., Lambourne L., Spirohn K., Begg B.E., Bian W., Brignall R., Cafarelli T., Campos-Laborie F.J., Charloteaux B. (2020). A reference map of the human binary protein interactome. Nature.

[5] Lund-Johansen F., Tran T., Mehta A. (2021). Towards reproducibility in large-scale analysis of protein–protein interactions. Nat. Methods.

[6] Hart G.T., Ramani A.K., Marcotte E.M. (2006). How complete are current yeast and human protein-interaction networks?. Genome Biol..

[7] Stumpf M.P., Thorne T., de Silva E., Stewart R., An H.J., Lappe M., Wiuf C. (2008). Estimating the size of the human interactome. Proc. Natl. Acad. Sci. USA.

[8] Rual J.F., Hirozane-Kishikawa T., Hao T., Bertin N., Li S., Dricot A., Li N., Rosenberg J., Lamesch P., Vidalain P.O. (2004). Human ORFeome version 1.1: A platform for reverse proteomics. Genome Res..

[9] Guo Y., Yu L., Wen Z., Li M. (2008). Using support vector machine combined with auto covariance to predict protein–protein interactions from protein sequences. Nucleic Acids Res..

[10] Pan X.-Y., Zhang Y.-N., Shen H.-B. (2010). Large-Scale prediction of human protein− protein interactions from amino acid sequence based on latent topic features. J. Proteome Res..

[11] Zhao X.-W., Ma Z.-Q., Yin M.-H. (2012). Predicting protein-protein interactions by combing various sequence-derived features into the general form of Chou’s Pseudo amino acid composition. Protein Pept. Lett..

[12] Sun T., Zhou B., Lai L., Pei J. (2017). Sequence-based prediction of protein protein interaction using a deep-learning algorithm. BMC Bioinform..

[13] Göktepe Y.E., Kodaz H. (2018). Prediction of protein-protein interactions using an effective sequence based combined method. Neurocomputing.

[14] Chen C., Zhang Q., Ma Q., Yu B. (2019). LightGBM-PPI: Predicting protein-protein interactions through LightGBM with multi-information fusion. Chemom. Intell. Lab. Syst..

[15] Tian B., Wu X., Chen C., Qiu W., Ma Q., Yu B. (2019). Predicting protein–protein interactions by fusing various Chou’s pseudo components and using wavelet denoising approach. J. Theor. Biol..

[16] Zhang L., Yu G., Xia D., Wang J. (2019). Protein–protein interactions prediction based on ensemble deep neural networks. Neurocomputing.

[17] Li F., Zhu F., Ling X., Liu Q. (2020). Protein Interaction Network Reconstruction Through Ensemble Deep Learning with Attention Mechanism. Front. Bioeng. Biotechnol..

[18] Czibula G., Albu A.-I., Bocicor M.I., Chira C. (2021). AutoPPI: An Ensemble of Deep Autoencoders for Protein–Protein Interaction Prediction. Entropy.

[19] Wang L., You Z.-H., Xia S.-X., Liu F., Chen X., Yan X., Zhou Y. (2017). Advancing the prediction accuracy of protein-protein interactions by utilizing evolutionary information from position-specific scoring matrix and ensemble classifier. J. Theor. Biol..

[20] Hashemifar S., Neyshabur B., Khan A.A., Xu J. (2018). Predicting protein–protein interactions through sequence-based deep learning. Bioinformatics.

[21] Gonzalez-Lopez F., Morales-Cordovilla J.A., Villegas-Morcillo A., Gomez A.M., Sanchez V. End-to-end prediction of protein-protein interaction based on embedding and recurrent neural networks. Proceedings of the 2018 IEEE International Conference on Bioinformatics and Biomedicine (BIBM).

[22] Richoux F., Servantie C., Borès C., Téletchéa S. (2019). Comparing two deep learning sequence-based models for protein-protein interaction prediction. arXiv.

[23] Li H., Gong X.-J., Yu H., Zhou C. (2018). Deep neural network based predictions of protein interactions using primary sequences. Molecules.

[24] Guo X., Liu R., Shriver C.D., Hu H., Liebman M.N. (2006). Assessing semantic similarity measures for the characterization of human regulatory pathways. Bioinformatics.

[25] Zhang S.-B., Tang Q.-R. (2016). Protein–protein interaction inference based on semantic similarity of gene ontology terms. J. Theor. Biol..

[26] Chen X.-W., Liu M. (2005). Prediction of protein–protein interactions using random decision forest framework. Bioinformatics.

[27] Zhang X., Jiao X., Song J., Chang S. (2016). Prediction of human protein–protein interaction by a domain-based approach. J. Theor. Biol..

[28] Maetschke S.R., Simonsen M., Davis M.J., Ragan M.A. (2012). Gene Ontology-driven inference of protein–protein interactions using inducers. Bioinformatics.

[29] Qi Y., Dhiman H.K., Bhola N., Budyak I., Kar S., Man D., Dutta A., Tirupula K., Carr B.I., Grandis J. (2009). Systematic prediction of human membrane receptor interactions. Proteomics.

[30] Thahir M., Sharma T., Ganapathiraju M.K. An efficient heuristic method for active feature acquisition and its application to protein-protein interaction prediction. Proceedings of the Great Lakes Bioinformatics Conference 2012.

[31] Goldberg D.S., Roth F.P. (2003). Assessing experimentally derived interactions in a small world. Proc. Natl. Acad. Sci. USA.

[32] Stark C., Breitkreutz B.-J., Reguly T., Boucher L., Breitkreutz A., Tyers M. (2006). BioGRID: A general repository for interaction datasets. Nucleic Acids Res..

[33] Park Y., Marcotte E.M. (2012). Flaws in evaluation schemes for pair-input computational predictions. Nat. Methods.

[34] Yu J., Guo M., Needham C.J., Huang Y., Cai L., Westhead D.R. (2010). Simple sequence-based kernels do not predict protein–protein interactions. Bioinformatics.

[35] Pinker E. (2018). Reporting accuracy of rare event classifiers. NPJ Digit. Med..

[36] Saito T., Rehmsmeier M. (2015). The precision-recall plot is more informative than the ROC plot when evaluating binary classifiers on imbalanced datasets. PLoS ONE.

[37] Ganapathiraju M.K., Thahir M., Handen A., Sarkar S.N., Sweet R.A., Nimgaonkar V.L., Loscher C.E., Bauer E.M., Chaparala S. (2016). Schizophrenia interactome with 504 novel protein-protein interactions. NPJ Schizophr..

[38] Consortium G.O. (2015). Gene ontology consortium: Going forward. Nucleic Acids Res..

[39] Huntley R.P., Sawford T., Mutowo-Meullenet P., Shypitsyna A., Bonilla C., Martin M.J., O’Donovan C. (2015). The GOA database: Gene ontology annotation updates for 2015. Nucleic Acids Res..

[40] Hunter S., Apweiler R., Attwood T.K., Bairoch A., Bateman A., Binns D., Bork P., Das U., Daugherty L., Duquenne L. (2009). InterPro: The integrative protein signature database. Nucleic Acids Res..

[41] Hulo N., Bairoch A., Bulliard V., Cerutti L., De Castro E., Langendijk-Genevaux P.S., Pagni M., Sigrist C.J. (2006). The PROSITE database. Nucleic Acids Res..

[42] Bateman A., Coin L., Durbin R., Finn R.D., Hollich V., Griffiths-Jones S., Khanna A., Marshall M., Moxon S., Sonnhammer E.L. (2004). The Pfam protein families database. Nucleic Acids Res..

[43] The UniProt Consortium (2021). UniProt: The universal protein knowledgebase in 2021. Nucleic Acids Res.

[44] National Center for Biotechnology Information. https://www.ncbi.nlm.nih.gov/.

[45] Mikolov T., Chen K., Corrado G., Dean J. (2013). Efficient estimation of word representations in vector space. arXiv.

[46] Altschul S.F., Madden T.L., Schäffer A.A., Zhang J., Zhang Z., Miller W., Lipman D.J. (1997). Gapped BLAST and PSI-BLAST: A new generation of protein database search programs. Nucleic Acids Res..

[47] Wen Z., Shi J., Li Q., He B., Chen J. (2018). ThunderSVM: A fast SVM library on GPUs and CPUs. J. Mach. Learn. Res..

[48] Paszke A., Gross S., Massa F., Lerer A., Bradbury J., Chanan G., Killeen T., Lin Z., Gimelshein N., Antiga L. (2019). Pytorch: An imperative style, high-performance deep learning library. Adv. Neural Inf. Process. Syst..

[49] Pedregosa F., Varoquaux G., Gramfort A., Michel V., Thirion B., Grisel O., Blondel M., Prettenhofer P., Weiss R., Dubourg V. (2011). Scikit-learn: Machine learning in Python. J. Mach. Learn. Res..

[50] Ke G., Meng Q., Finley T., Wang T., Chen W., Ma W., Ye Q., Liu T.-Y. (2017). Lightgbm: A highly efficient gradient boosting decision tree. Adv. Neural Inf. Process. Syst..

[51] Rodriguez J.J., Kuncheva L.I., Alonso C.J. (2006). Rotation forest: A new classifier ensemble method. IEEE Trans. Pattern Anal. Mach. Intell..

[52] Li Y., Ilie L. (2017). SPRINT: Ultrafast protein-protein interaction prediction of the entire human interactome. BMC Bioinform..

[53] Jia J., Li X., Qiu W., Xiao X., Chou K.-C. (2019). iPPI-PseAAC (CGR): Identify protein-protein interactions by incorporating chaos game representation into PseAAC. J. Theor. Biol..

[54] Shen J., Zhang J., Luo X., Zhu W., Yu K., Chen K., Li Y., Jiang H. (2007). Predicting protein–protein interactions based only on sequences information. Proc. Natl. Acad. Sci. USA.

[55] Dubchak I., Muchnik I., Holbrook S.R., Kim S.-H. (1995). Prediction of protein folding class using global description of amino acid sequence. Proc. Natl. Acad. Sci. USA.

[56] Du X., Sun S., Hu C., Yao Y., Yan Y., Zhang Y. (2017). DeepPPI: Boosting prediction of protein–protein interactions with deep neural networks. J. Chem. Inf. Model..

[57] Jia J., Liu Z., Xiao X., Liu B., Chou K.-C. (2015). iPPI-Esml: An ensemble classifier for identifying the interactions of proteins by incorporating their physicochemical properties and wavelet transforms into PseAAC. J. Theor. Biol..

[58] Zhang Z.-H., Wang Z.-H., Wang Y.-X. A new encoding scheme to improve the performance of protein structural class prediction. Proceedings of the International Conference on Natural Computation.

[59] Yu B., Chen C., Wang X., Yu Z., Ma A., Liu B. (2021). Prediction of protein–protein interactions based on elastic net and deep forest. Expert Syst. Appl..

[60] Yang L., Xia J.-F., Gui J. (2010). Prediction of protein-protein interactions from protein sequence using local descriptors. Protein Pept. Lett..

[61] You Z.-H., Chan K.C., Hu P. (2015). Predicting protein-protein interactions from primary protein sequences using a novel multi-scale local feature representation scheme and the random forest. PLoS ONE.

[62] You Z.-H., Zhu L., Zheng C.-H., Yu H.-J., Deng S.-P., Ji Z. (2014). Prediction of protein-protein interactions from amino acid sequences using a novel multi-scale continuous and discontinuous feature set. BMC Bioinform..

[63] Ding Y., Tang J., Guo F. (2016). Predicting protein-protein interactions via multivariate mutual information of protein sequences. BMC Bioinform..

[64] Chou K.C. (2001). Prediction of protein cellular attributes using pseudo-amino acid composition. Proteins Struct. Funct. Bioinform..

[65] Chou K.-C. (2000). Prediction of protein subcellular locations by incorporating quasi-sequence-order effect. Biochem. Biophys. Res. Commun..

[66] Chen M., Ju C.J.-T., Zhou G., Chen X., Zhang T., Chang K.-W., Zaniolo C., Wang W. (2019). Multifaceted protein–protein interaction prediction based on Siamese residual RCNN. Bioinformatics.

[67] Guo Y., Li M., Pu X., Li G., Guang X., Xiong W., Li J. (2010). PRED_PPI: A server for predicting protein-protein interactions based on sequence data with probability assignment. BMC Res. Notes.

[68] Liu L., Cai Y., Lu W., Feng K., Peng C., Niu B. (2009). Prediction of protein–protein interactions based on PseAA composition and hybrid feature selection. Biochem. Biophys. Res. Commun..

[69] Martin S., Roe D., Faulon J.-L. (2005). Predicting protein–protein interactions using signature products. Bioinformatics.

[70] Zhou Y.Z., Gao Y., Zheng Y.Y. (2011). Prediction of protein-protein interactions using local description of amino acid sequence. Advances in Computer Science and Education Applications.

[71] Yao Y., Du X., Diao Y., Zhu H. (2019). An integration of deep learning with feature embedding for protein–protein interaction prediction. PeerJ.

[72] Mohamed T.P., Carbonell J.G., Ganapathiraju M.K. (2010). Active learning for human protein-protein interaction prediction. BMC Bioinform..

