# Supplementary material for: Benchmark Evaluation of Protein–Protein Interaction Prediction Algorithms
Source: Molecules. 2021 Dec 22;27(1):41. doi: 10.3390/molecules27010041 (PMC8746451; doi:10.3390/molecules27010041)
Supplement: Supplementary file 1 [file molecules-27-00041-s001.zip › molecules-1473408-supplementary.pdf]

Supplementary File 1:

Benchmark Evaluation of Protein Interaction Prediction Algorithms

Brandan Dunham and Madhavi K. Ganapathiraju

S1: Human Protein-Protein Interaction Preprocessing

The list of positive protein interactions utilized for our various tests was curated from The Biological General Repository for Interaction Datasets (BioGRID, v4.4.198, compiled May 2021) [1]. Only protein interactions from BioGRID matching as direct interactions, as defined by the Molecular Interactions Controlled Library (term MI:0407) and its descendants were kept as valid protein interactions. All provided Entrez IDs from BioGRID were mapped through NCBI's gene history to ensure we used up to data gene and protein identifiers (downloaded May 2021) [2]. Additionally, filtering was done to remove interactions involving non-human proteins (9375), self-interactions (4084), protein-RNA bindings (7016), non-UniProt proteins (1953), and duplicate interactions (32875), to create a final list of 123642 unique, human, protein-protein interactions. After removing proteins with a sequence of less than 30 amino acids in UniProt, we obtained our final list of 123626 protein interactions among 14678 proteins. A total of 19115 human Entrez IDs were mapped to UniProt, creating our full list of proteins to generate random pairs from.

For plots and analysis comparing HIPPIP, Qi's Membrane Receptors, and SPRINT, processing of true protein-protein interactions was done similarly to the original HIPPIP paper [3–5]. The primary differences between the data used for these analyses and data used for other tests in our work are:

1. When filtering BioGRID data, no filtering based on UniProt or sequence length is done, yielding a final list of 125464 interacting protein pairs.
2. As done in the original High-Precision Protein-Protein Interaction Prediction (HiPPIP) paper, data from HPRD was also used [6]. To map HPRD identifiers to Entrez, we relied on mapping to NCBI's Homo_sapiens.gene_info and gene2accession files [2]. Identifiers were mapped to Gene Info Symbols, Gene Accession's RNA nucleotide, Protein Accession, and Genomic Nucleotide numbers, Gene Info Symbols from nomenclature authority, and Gene Info Synonyms, in descending order of priority. From the 37060 binary, unique, non-self-interacting protein interactions provided by HPRD, we generated 36853 interactions using Entrez Identifiers. We note that our original conversion of HPRD data was done in 2018, using NCBI files from 2018 during the conversion process. However, as HPRD has not been updated since 2010, simply mapping these values using NCBI's gene history files (to ensure we have the most up to data IDs) should provide similar results to using NCBI files from 2021.

Combining the previously mentioned HPRD and BioGRID datasets, our final set positive interaction contained 143535 unique protein interactions. Predictions from SPRINT and Qi were downloaded from their respective published sets, and mapped from UniProt symbols and identifiers to Entrez [4,5]. For Qi's and HiPPIP data, only predictions suggested as positive scores were utilized when creating our plots.

For our final plots comparing HiPPIP to Held Out methods, HiPPIP utilized a different test set than the Held Out methods. Additionally, HiPPIP is a pre-trained model, and the training data for HiPPIP was unknown at the time of this analysis, thus the proteins were not held out and the test data may have overlapped with the training data. The test data

used for HiPPIP evaluations consisted of 300 positives interactions from BioGRID and 99700 random pairs per set, with 10 sets used per test, from either all current interactions (HiPPIP-a), or from interaction data from January 2017 or prior (BioGRID 3.4.145, HiPPIP-b). When creating these plots, HPRD interactions in the test datasets of both methods were additionally marked as positive when calculating results. This did not affect the training data or training process of the Held Out methods, which were still trained with only BioGRID interactions, but does mean the percentage of data with a positive class label is slightly above 0.3% when performing all tests.

All UniProt to Entrez mappings, for protein interactions, Gene Ontology annotations, and all other data coded with UniProt identifiers, were done using UniProt's Human ID Mapping file (downloaded in February and June 2021). We note that some of our original mapping of proteins between UniProt and Entrez to create the initial sequences and generate datasets was done with files downloaded in February 2021. Over the time this project was run, 3 of these proteins were marked as obsolete or withdrawn, and thus, by the time mappings were created for annotation-based features using June 2021 mappings, 3 proteins were not used when mapping annotations to genes (and thus had no annotations). These 3 proteins did not appear in any known interactions, and we believe the influence of this difference is minimal (as it is unlikely they would have had many, if any annotations given they have been removed from the NCBI gene list). Thus, while 19115 proteins with sequences were used, only 19112 Entrez Genes were mapped to UniProt for annotations.

S2: Implementation of Sequence-Based Features

In this section we describe the computations for a total of 26 different calculations for converting sequences into machine learning features. We primarily followed prior work describing the formulas for each calculation, and, when necessary, attempted to normalize the final feature vectors in a way that kept all the values close to a 0 to 1 or -1 to 1 range. This was done to ensure that when using multiple features, their value ranges would be similar. Any unclear explanations in previous works, or deviations from formulas listed in previous works, will be noted here. Additionally, we note which algorithms we have implemented use which features. For more notes on the implemented algorithms, see Appendix E.

Note: When using if statements in equations, we followed the following formula, where the condition is inside of parenthesis followed by values if the condition is met or not met.

$$If\ (condition): "True\ value"\ else\ "False\ value"$$

Amino Acid Count Features:

Features in this section are primarily based on counting the occurrences of different amino acids, or combinations of amino acids.

Amino Acid Composition (AAC): Count of each type of all amino acids in sequence, divided by the total number of amino acids, as shown in Equation S1. Creates 20 features per protein sequence.

$$AAC_x = \frac{\sum_{n=0}^{len(Seq)} 1\ if\ Seq_n == x}{len(Seq)} \tag{S1}$$

Used by: Ding 2016[7], Du 2017[8], Jia 2019[9]

Dipeptide Composition: Uses the same formula as AAC but counts pairs of amino acids instead of individual Amino Acids. Produces 400 features per protein sequence.

Used by: Du 2017[8]

Conjoint Triad Method (CT): Groups amino acids by dipole and side chain volumes into 7 distinct groups and computes the count of each set of 3 consecutive groups. Groups are normalized by subtracting the minimum value and dividing by the maximum value, as shown in Equations S2, S3. Creates 343 (7^3) features per protein sequence.

$$Count_x = \sum_{n=0}^{len(Seq)-2} if\ (group(Seq_n), group(Seq_{n+1}), group(Seq_{n+2})) == x): 1\ else\ 0 \tag{S2}$$

$$CT_x = \frac{Count_x - (\forall z \min(Count_z))}{\forall z \max(Count_z)} \tag{S3}$$

Used by: Pan 2010 LDA[10], Sun 2017 CT[11], Li 2020[12], Czibula 2021[13]

Weighted Skip-Sequential Conjoint Triad: Combines the regular conjoint triad using 3 consecutive amino acids with a conjoint triad calculation using 4 consecutive amino acids. When using 4 amino acids, 1 of the 2 middle acids is skipped, creating 2 separate fragments of length 3. The new counts are combined with the original and normalized, as shown in Equations S4–S8. **Note:** The original paper using weighted skip-sequential conjoint triad did not mention normalization. To keep the values small and similar to other features, we used the same normalization as the original conjoint triad method. Additionally, no weight was suggested for the skip-sequential triads, only that they were lower weighted than consecutive conjoint triad counts. In our work, we used a weight of 0.5 for skip sequential triads.

$$Org\ Count_x = \sum_{n=0}^{len(Seq)-2} if\ (group(Seq_n), group(Seq_{n+1}), group(Seq_{n+2})) == x): 1\ else\ 0 \tag{S4}$$

$$Skip\ 1\ Count_x = \sum_{n=0}^{len(Seq)-3} if\ (group(Seq_n), group(Seq_{n+2}), group(Seq_{n+3})) == x): 1\ else\ 0 \tag{S5}$$

$$Skip\ 2\ Count = \sum_{n=0}^{len(Seq)-3} if\ (group(Seq_n), group(Seq_{n+1}), group(Seq_{n+3})) == x): 1\ else\ 0 \tag{S6}$$

$$All\ Count = Org\ Count + w * (Skip\ 1\ Count) + w * (skip\ 2\ Count) \tag{S7}$$

$$SWCT_x = \frac{All\ Count_x - (\forall z \min(All\ Count_z))}{\forall z \max(All\ Count_z)} \tag{S8}$$

Used by: Göktepe 2018[14]

Multivariate Mutual Information (MMI): MMI computes the probabilities of amino acids grouped using the same 7 groups as the Conjoint Triad method. MMI analyzes consecutive amino acids at lengths (L) of 1, 2, and 3, computing the mutual information of each. MMI computes groups such as amino acids sequence (b,a) is grouped with amino acids sequence (a,b), and thus creates 7 L=1 features, 28 L=2 features, and 84 L=3 features. Computation of the features I_a, I_{ab}, and I_{abc} can be found in equations S9–S12, where a, b, and c represent amino acid groups and m represents a set of CT groups of length 1, 2, or 3 [7].

$$Freq_m = \frac{1}{len(Seq)+1} + \sum_{n=0}^{len(Seq)-len(m)+1} if\ (group(Seq_n) \ldots group(Seq_{n+len(m)-1}) = m): \frac{1}{len(Seq)+1}\ else\ 0 \tag{S9}$$

$$I_a = Freq_a \tag{S10}$$

$$I_{ab} = Freq_{ab} * \ln\left(\frac{Freq_{ab}}{Freq_a * Freq_b}\right) \tag{S11}$$

$$I_{abc} = I_{ab} + \frac{Freq_{ac}}{Freq_c}\ln\left(\frac{Freq_{ac}}{Freq_c}\right) - \frac{Freq_{abc}}{Freq_{bc}}\ln\left(\frac{Freq_{abc}}{Freq_{bc}}\right) \tag{S12}$$

Used by: Ding 2016[7]

Composition, Transition, Distribution (CTD): CTD features are a concatenation of three different types of features on grouped sets of amino acids. First, all amino acids are split into groups. Most commonly, the 7 groups from the Conjoint Triad method are used. However, some implementations utilize as few as 3 groups based on different criteria or use 20 groups to represent the 20 amino acids. After grouping into N groups, each of the calculations can be computed.

Composition: Composition is equivalent to Amino Acid Composition, using N groups instead of all 20 amino acids. Produces N features per amino acid sequence

Transition: Transition uses a similar formula to Dipeptide Acid composition. However, this method only counts consecutive amino acids that are not in the same group, and counts groups symmetrically, such as an amino acid pair belonging to groups (1,2) or groups (2,1) are counted the same. Produces $\frac{N^2-\mathrm{N}}{2}$ features per amino acid sequence.

Distribution: Distribution records the distances along the amino acid sequence at which the group occurs. For each group, the index of the amino acid representing the 25th, 50th, and 75th percentile of the group's representation in the amino acid sequence, along with the first and last occurrence of the group, are recorded. These values are divided by the length of the amino acid sequence to convert to sequences. Distribution creates N*5 features.Thus, CTD creates $N * 6 + \frac{N^2-\mathrm{N}}{2}$ features, given N groups.

Note: In the original paper, all values were represented as percentages, however the inputs used for the learning algorithm appeared to scale the 0-100 range. We kept these features in the range 0-1, to be more similar to other features. Additionally, to find 25th, 50th, and 75th percentiles, we rounded to the closest instance of the given group.

For example, to find the 25th percentile from a group that occurs 8 times, round((8-1)*.25) = 2, thus the 3rd instance (0 indexed) would be used for calculating the 25th percentile value. The value would be the position of this instances in the sequence, divided by the sequence length -1.

Used by: Du 2017[8] (Du used various physicochemical features to create 24 different groupings, with 3 groups each (N=3), to compute 24 * 21 = 504 CTD features).

Descriptor Features:

Descriptor Features break amino acid sequences into various fragments and run computations across the each individual or combination of fragments created from the amino acid sequence. These methods commonly use an amino acid count feature, such as CTD, on each fragment and fragment combination they create.

Local Descriptor (LD): Local Descriptor encoding breaks a protein's amino acid sequence into 4 equal length parts. From these parts, 9 segments are formed from all possible sets of consecutive fragment groups that do not contain the whole sequence. Thus, 4 groups (g1, g2, g3, g4) are 1/4th the length of the protein, 3 groups (g12, g23,

g34) are 1/2th the length of the protein, 2 groups (g123, g234) are 3/4th the length of the protein. A final group containing the middle 75% of amino acids in the protein sequence comprises the 10th and final subsequence. For all 10 subsequences, CTD using Conjoint Triad groups is computed, creating 63*10 = 630 features.

Used by: Zhou 2011[15]*, Chen 2019[16], Zhang 2019[17]*, Li 2020[12]*

***Note:** Due to a bug in the initial implementation, the 10th subsequence used the entire amino acid sequence, instead of the middle 75%, for these 3 algorithms.

Multi-scale Local Descriptor (MLD) Encoding: MLD encoding works similarly to LD encoding. The protein sequence is split into N parts, and all consecutive regions of the N parts are used as fragments for the protein. This method allows for a flexible N, and setting N=4 would yield the first 9 segments of the LD method, excluding only the final fragment composed of the middle 75%. Like the LD encoding method, all fragments are run through CTD using Conjoint Triad groups, yielding $(\frac{N^2+\mathrm{N}}{2}-1) * 63$ features.

Used by: You 2015[18] (with N= 4)

Multi-scale Continuous and Discontinuous Local Descriptor (MCD) Encoding: MCD encoding works almost the same as MLD encoding, with the lone exception being that it does not remove discontinuous sets of fragments. Thus, all combinations of groups, except for all groups and no groups, are used. If splitting into 4 parts, fragment groups such as (g14, g134) would be valid, despite having gaps. Finally, all fragments are run through CTD using Conjoint Triad groups, yielding $(2^N - 2) * 63$ features.

Used by: Zhang 2019[17] (used with N=5)

Encoding Based on Grouped Weight (EGBW): EGBW splits a sequence into N subsequences, where the start of each subsequence is at the beginning of the protein's sequence, and the ending points are equally spaced along the protein's sequence. The 20 amino acids are clustered into 4 groups based on hydrophobicity and charge (g1=(GAVLIMPFW), g2=(QNSTYC), g3=(DE), g4=(HKR)). From these 4 groups, 3 unique sets of splits are created, each split containing 2 groups. Each split is assigned a 0 or 1 value, which is then used to encode the sequence in 3 different ways. For example, given groups g1, g2, g3, and g4, the first split would have groups (g1, g2) =1, the second split would have groups (g1, g3) = 1, and the third split would have groups (g1, g4) = 1, with the remaining groups set to 0. These splits then make 3 binary encodings of the full protein sequence. On each of the N subsequences, for each of the 3 binary encodings, the percentage of values equal to one creates the new features. Thus, N*3 features are created using the EGBW method.

Used by: Tian 2019[19] (N=11)

Physicochemical Based Features:

Physicochemical based features use the numeric values of various calculations of amino acid properties, such hydrophobicity and side-chain mass, to compute numeric values for entire amino acid sequences. Traditionally, these features follow a simple set of steps. First, a group of amino acid properties is selected, and these properties are normalized such that each property has a mean of 0, and a standard deviation of 1. Secondly, the amino acid sequence is encoded with these values in place of the standard letters used for amino acids. Thirdly, optionally, the encoded sequence is normalized by subtracting its mean. Finally, for all values m=1 to max lag, each pair of amino acids along the sequence that are m distance apart have a value calculated based on the physicochemical properties. The average of these values for each lag and each physicochemical property are used as features to represent the protein. These initial equations required by various physicochemical feature-based implementations are listed in Equations S13–S17.

Note: For our computations, non-standard amino acids were removed from sequences, leaving only the 20 standard amino acids prior to running these calculations. This was necessary as most physiochemical properties only list values for the standard 20 amino acids. Removing these values may differ from ways other methods may have handled them, such as assign 0 values, or average values, to all non-standard amino acids. Occurrences of non-standard amino acids was rare within all datasets.

Note 2: For any physicochemical based calculations that did not explicitly list what features were used, we assumed the feature set was the same used by Guo et al. 2008 [20]. (hydrophobicity, hydrophicility, volumes of side chains of amino acids, polarity, polarizability, solvent-accessible surface area (SASA), and net charge index of side chains of amino acids)

Note 3: In Guo et al.'s original supplementary data, a value was typed as 2 when in literature it is listed as 0.2 (amino acid N, feature set HOPT810101). We use the corrected value (0.2) in all our computations.

$$Mean\ Normalized\ Property\ (MNP)_{aa} = Property_{aa} - \frac{\sum_{\forall z} Property_z}{\#\ amino\ acids} \tag{S13}$$

$$Normalized\ Property\ (NP)_{aa} = \frac{MNP_{aa}}{\sqrt{\sum_{\forall z} \frac{{MNP_z}^2}{\#\ amino\ acids}}} \tag{S14}$$

$$Encoded\ Sequence\ (ES) = NP_x \quad \mathrm{x} \in \mathrm{Seq} \tag{S15}$$

$$Normalized\ Encoded\ Sequence\ (NES) = ES_x - \sum_{\forall z} \frac{ES_z}{len(NES)} \quad \mathrm{x} \in \mathrm{ES} \tag{S16}$$

$$Avg\ Squared\ NES\ (AvgSqNES) = \frac{\sum_{\forall z} {NES_z}^2}{len(Seq)} \tag{S17}$$

Autocovariance (AC): Autocovariance computes the average of the product of lag distance amino acids using a normalized protein sequence (Equation S18). Computes number of properties * max lag features.

$$AC_{\mathrm{property},lag} = \sum_{\mathrm{x}=0}^{len(Seq)-lag} \frac{NES_x * NES_{x+lag}}{(len(Seq) - lag)} \tag{S18}$$

Used by (with standard 7 properties and max lag = 30): Guo 2008[20], Pan 2010 AC[10], Sun 2017 AC[11], Zhang 2019[17], Li 2020[12]

Used by (with non-standard lag): Tian 2019[19] (max lag=11)

Used by (with non-standard properties): Czibula 2021[13] (14 properties)

Normalized Moreau-Broto Autocorrelation (NMBA): Normalized Moreau Broto computes the average of products of lag distance amino acids using an unnormalized sequence (Equation S19). Computes number of properties * max lag features.

$$NMBA_{\mathrm{property},lag} = \sum_{\mathrm{x}=0}^{len(Seq)-lag} \frac{ES_x * ES_{x+lag}}{(len(Seq) - lag)} \tag{S19}$$

Used by: Zhao 2012[21] (8 properties), Ding 2016[7] (6 properties), Chen 2019[16] (max lag=9)

Moran Autocorrelation: Moran calculates the average of products of lag distance amino acids using normalized sequences and divides these averages by the average of the squared normalized sequence, as shown in Equation S20. Computes number of properties * max lag features.

$$Moran_{\text{property},lag} = \sum_{x=0}^{len(Seq)-lag} \frac{NES_x * NES_{x+lag}}{(len(Seq) - lag) * AvgSqNES} \tag{S20}$$

Used by: Zhao 2012[21] (8 properties), Chen 2019[16] (max lag=9)

Geary Autocorrelation: Geary calculates half of the average of the squared difference between lag distance amino acids using unnormalized sequences, and divides these average by the average of the squared normalized sequence, as show in Equation S21. Computes number of properties * max lag features.

$$Geary_{\text{property},lag} = \sum_{x=0}^{len(Seq)-lag} \frac{(ES_x - ES_{x+lag})^2}{2 * (len(Seq) - lag) * AvgSqNES} \tag{S21}$$

Used by: Zhao 2012[21] (8 properties), Chen 2019[16] (max lag=9)

Average Squared (AvgSq): Average Squared computes the average of the squared difference between lag distance amino acids using unnormalized sequences (Equation S22). Computes number of properties * max lag features.

$$AvgSq_{\text{property},lag} = \sum_{x=0}^{len(Seq)-lag} \frac{(ES_x - ES_{x+lag})^2}{(len(Seq) - lag)} \tag{S22}$$

Discrete Wavelet Transform Physicochemical (DWTP): Discrete Wavelet Transform Physicochemical replaces all values within each protein's amino acid sequence with physicochemical properties (Encoded Sequence), which are then run through a discrete wavelet transforms (wavelet type = db1, levels=4). For each property, the min, max, average, and standard deviation are calculated from the values returned by DWT, computing 4 * property features per protein.

Used by: Jia 2015[22]

Pairwise Physicochemical Based Features:

Pairwise physicochemical based features work the same are the original physicochemical based features, except instead of subtracting or multipling features for a single amino acid, they rely on calculated matrices of properties between pairs of features. **Note:** When sequence order descriptors were introduced by Chou[23], the matrix created by Schneider and Wrede[24] had been normalized such that each row of values was mapped between 0 and 1. Many authors also use Grantham's chemical distance table[25], and could use other tables that are not similarly normalized. We subsequently applied 0-1 normalization per row to any feature tables used for pairwise physicochemical properties, to match the normalization used by this original table.

Sequence Order (SqOr): Sequence order calculates the average squared values of pairwise physicochemical properties (Equation S23). Computers number of properties * max lag features. (Default 2 properties, max lag=30). **Note:** Previous literature summed the values, we used an average to keep the values in the number range near 0-1).

$$SqOr_{\text{property},lag} = \sum_{\text{x}=0}^{len(Seq)-lag} \frac{(Norm\_Prop(Seq_x, Seq_{x+lag})^2}{(len(Seq) - lag)} \tag{S23}$$

Used By: Zhao 2012[21], Du 2017[8]

Concatenation of Amino Acid Count and Physicochemical Features:

Features in this concatenation category typically use two or more of the previously mentioned calculations, which are then combined into a single set of features.

Pseudo Amino Acid Count (PSAAC): Pseudo Amino Acid Count computes and concatenates both Amino Acid Count (AAC) and Average Squared (AvSq) {Chou, 2001 #176}. If multiple properties are used for multiple Average Squared calculations, the returned vectors are averaged (Avg AvSq). The averaged Average Squared values are the multiplied by a user defined weight, and final feature vector is then created by concatenation and normalized, as shown in Equations S24, S25. Amino acid count is normalized prior to concatenating with the Averaged Average Squared (Avg AvSq) vector. Creates 20 + max lag features. By default, 3 physicochemical properties are used (hydrophobicity, hydrophilicity, and side-chain mass), with a weight of 0.1.

$$Avg\ AvgSq_x = weight * \frac{\sum_{z=0}^{num\_properties} AvgSq_x}{num\_properties} \tag{S24}$$

$$PSAAC = \frac{concat(AAC, Avg\ AvgSq)}{\sum_{z=0}^{z=20+max\ lag} concat(AAC, Avg\ AvgSq)} \tag{S25}$$

Used by: Pan 2010 PSAAC (max lag=20)[10], Zhao 2012 (max lag=30)[21], Göktepe 2018 (max lag=20)[14], Chen 2019 LGBM (max lag=3)[16], Tian 2019 (max lag=9)[19], Li 2020 (max lag=15)[12]

Amphiphilic Pseudo Amino Acid Count (APSAAC): Amphiphilic Pseudo Amino Acid Count uses the same final formula as PSAAC but does not average together values over different properties. Thus, for K properties, 20 + k * max lag features are generated. The full formula is shown in Equation S26. Amino acid count is normalized prior to concatenating with the k * max lag values generated by the physicochemical properties. By default, 2 properties are used, with a user-defined weight of 0.5. **Note:** in order to keep the values for APSAAC similar to PSAAC, we used a weight of 0.05, as this would be more equivalent to the balance of AAC and AvSq values found in the original formula We also note that in the original paper by Chou, regarding the value of w for their study *"w* can be of course assigned with other values, but this would not make a significant difference to the final results" [26].

$$APSAAC = \frac{concat(AAC, weight * AvgSq_{1...k})}{\sum_{z=0}^{z=20+max\ lag*k} concat(AAC,\ weight * AvgSq_{1...k})} \tag{S26}$$

Used by: Du 2017 (max lag=30)[8]

Quasi-Sequence Order (Quasi): Quasi sequence order follows a similar format as both PSAAC and APSAAC. However, Quasi-Sequence Order uses the Sequence Order (SqOr) feature based on a pairwise matrix of amino acid properties rather than the Average Squared (AvSq) formula. This formula (Equation S27) has only been defined for using a single property, with a different set of computations done for different properties. Thus, (20+lag) features are computed for each property. By default, max lag=30, weight=0.1, and the formula is computed for 2 different properties.

$$Quasi_{property} = \frac{concat(AAC, weight * SqOr)}{\sum_{z=0}^{z=20+max\ lag} concat(AAC,\ weight * SqOr)} \quad \text{(S27)}$$

Used by: Zhao 2012[21], Du 2017[8]

Position-Specific Scoring Matrices (PSSM) Features:

PSSM features are based on evolutionary calculation of amino acids across various species and proteins. For each protein, we computed PSSMs using the PSI-BLAST algorithm, with all proteins in UniProt's SwissProt database serving as the set of proteins queried against, with 3 iterations and a significance value of 0.001 set as hyperparameters for the algorithm.[27,28] When no matches were found using PSI-Blast and SwissProt, all proteins were encoded using BLOSUM62 values per amino acid.

PSSM: PSSM features are simply the raw Position-Specific Scoring Matrices, as computed from PSI-BLAST.

Used by: Hashemifar 2018[29]

PSSM Dipeptide Composition (DPC)/Bi-Gram: Using PSSMs, the DPC/Bi-Gram method can be computed by multiplying, for all adjacent pairs of amino acids, the values for each pair of amino acids. These values are then averaged, computing 400 features, one for each pair of amino acids. The formula is shown in Equation S28.

$$PSSM\ DPC_{a,b} = \frac{\sum_{i=0}^{i=len(Seq)-1} PSSM(i,a) * PSSM(i+1,b)}{len(Seq)-1} \quad \text{(S28)}$$

Used by: Göktepe 2018[14]

PSSM Discrete Cosine Transform (PSSMDCT): PSSM DCT features are computed by running a multidimensional discrete cosine transform on each protein's PSSM (dctType=2, dctNormType=ortho). By default, the top 400 values are kept as features. **Note:** We assumed top 400 values would be the 20x20 values in the top left of the returns DCT values, as literature suggests the top left values are the most informative and contain most of the information.

Used By: Wang 2017[30]

Other Features:

Chaos Game Representation: Chaos Game Representation features are computed by first transforming the amino acid sequence into nucleotides using a pre-set table mapping each amino acid to a single triplet of nucleotides. From there, each nucleotide is assigned the value of a different corner of a unit length box ((1,1), (1,0), (0,0), (0,1)), and a plot is made on a 2D-grid space representing the protein sequence. Starting from the center (0.5,0.5), the nucleotide sequence is iterated in order, with new points being generated on the plot halfway between the current point and the new nucleotide. Ideally, this will generate a pattern representing the structure of the protein sequence. The 2D-grid is finally broken into smaller boxes, with the number of points per box being recorded as features. **Note:** Keeping in line with other features, we normalized these counts by dividing by the length of the protein sequence.

Used by: Jia 2019[9] (4 x 4 grid, 16 features)

One-hot / Numeric Encoding: Numeric encoding simply replaces each amino acid within an amino acid sequence with a unique number, such as the numbers 1-20 for the 20 different standard amino acids. One-hot

encoding similarly encodes a sequence, but uses a binary vector, where, for the 20 standard amino acids, would consist of a unique set of 20 binary values per amino acid, with 19 of the values for each amino acid being 0.

In literature, the length/number of values varies slightly depending on the implementation, as some algorithms encoded only the 20 standard amino acids, whereas other encoded 22 or 23, and some lumped all remaining amino acids into a final 'any' value. When no 'any' value was present, any non-standard/non-listed amino acids were dropped from the amino acid sequence prior to creating the feature.

One-hot Encoding Used by: Chen 2019 RNN (7 features per amino acid, conjoint triad grouping)[31], Richoux 2019 (24 features per amino acid, including an any grouping)[32]

Numeric Encoding Used by: Gonzalez-Lopez 2019 (encoding 8000 features per 3 amino acids, using non-overlapping windows of length 3, with the first amino acids truncated if necessary)[33], Li 2020 (22 features per amino acid, **Note:** we assumed U and O were the nonstandard amino acids used)[12]

Skip-Gram: Skip -Gram models create feature vectors per word using a neural network. Given a word for training, and a set of neighboring words to predict, the skip-gram model attempts to output the most likely words neighboring a given word [34]. As the number of possible words may be large, training is commonly done using negative sampling, where only a random subset of all possible words are used as negatives for each piece of training data. To create skip-gram representations, the sequence is first encoded using a numeric encoding method. Next, each amino acid is treated as a word, and neighboring (window) amino acids are treated as targets, to train the network. Finally, the embeddings for the encoding layer of the network are used to create a numeric vector per amino acid.

For our training purposes, only sequences utilized by the protein interaction datasets were used for training each skip-gram model. By default, we trained the skip-gram model for 1 epoch over all sequence data in a given dataset, with a negative sampling size of 5, using Stochastic Gradient Descent (SGD) with a learning rate of 0.01. All models were implemented in PyTorch [35].

Used by: Chen 2019 RNN (generated 5 features per amino acid, using a window size of 7 and negative sampling size of 5)[31], You 2019 (generated 20 features per amino acid, using a window size of 4. **Note:** Uses 25 unique amino acids. We assumed B, Z, U, O, and X were the nonstandard values)

S3: Non-sequence/Annotation-Based Features

In this section, we describe the computation for pairwise and other non-sequence-based features used to encode protein pairs for protein interaction prediction. Please note that if information for a given feature does not exist, all pairwise scores involving that feature for protein pairs containing the given protein were scored as 0.

Note, that several features based on annotations contain many annotations per protein. When handling such features, the common method is to compute scores for all pairs of annotations between the pair of proteins, creating an MxN grid of scores for each possible pair. To reduce this grid to a single value, one of the following formulas is used:

$$Sum = \sum_{m=1}^{M} \sum_{n=1}^{N} Val(m,n) \quad \text{(S29)}$$

$$Average = \frac{Sum}{M * N} \quad \text{(S30)}$$

$$Max = max_{m\in(1\ldots M),n\in(1\ldots N)} Val(m,n) \quad \text{(S31)}$$

$$Product\ (Prod) = 1 - \prod_{m=1,n=1}^{M,N} 1 - Val(m,n) \quad \text{(S32)}$$

$$Best\ Matching\ Average\ (BMA) = \sum_{m=1}^{M} \frac{max_{n\in(1\ldots N)} Val(m,n)}{2*M} + \sum_{n=1}^{N} \frac{max_{m\in(1\ldots m)} Val(m,n)}{2*N} \quad \text{(S33)}$$

Semantic Similarity Features:

Semantic similarity features compute the similarity of various terms within a hierarchal structure. In this case, the structure is the Gene Ontology hierachy. Most semantic similarity measures based on the Gene Ontology hierarchy rely on the information content of the most informative common ancestor (MICA). Information content (IC) is computed based on the rarity of any protein being annotated with a given GO term or any of its descendants. For our calculations, IC was computed by the GOATools library [36]. For each pair of terms, the MICA is computed as the term with the largest information content that is also an ancestor between both terms.

A list of common computations used by semantic similarity are given in Equations S34–S39. Semantic similarity is computed for each ontology (molecular function, biological process, cellular component) individuallly, for each pair of annotations, and aggregated using one of the 5 previously mention aggregations (Equations S29–S33), to compute 3 final feature values. Please note that Product aggregation may be invalid for some semantic similarity measurements, such as Resnik, since the values produced by these formulas do not fall between 0 and 1.

$$ancestors(annos)_{anno} = list\ of\ all\ GO\ terms\ that\ are\ ancestors\ of\ anno, including\ anno \quad \text{(S34)}$$

$$Information\ Content\ (IC)_{term,annos} = -\log\left(\frac{\sum_{t\,\in\,annos} 1\ if\ term \in ancestors(t)}{len(annos)}\right) \quad \text{(S35)}$$

$$MICA_{a,b} = term(t)\ such\ that\ IC_t = max_{t\,\in\,ancestor(a)\&ancestor(b)}(IC_t) \quad \text{(S36)}$$

$$Descendent\ IC\ (DescIC)_a = max_{t\,\in\,descendents(a)}(IC_t) - IC_a \quad \text{(S37)}$$

$$MICD_{a,b} = term(t)\ such\ that\ IC_t = max_{t\,\in\,descendents(a)\&descendents(b)}(IC_t) \quad \text{(S38)}$$

$$Dist_{a,b} = minimum\ edge\ distance\ between\ terms\ a\ and\ b \quad \text{(S39)}$$

Resnik Semantic Similarity: Computes similarity based on the IC value of the MICA term (Equation S40) [37].

$$Rensik_{a,b} = IC(MICA_{a,b}) \quad \text{(S40)}$$

Used by: Gou 2006 (max)[38], Zhang 2016 (BMA)[39], Simple Ensemble (BMA)

Lin Semantic Similarity: Computes similarity based on the IC values of the MICA term and the given terms a and b (Equation S41)[40].

$$Lin_{a,b} = \frac{2 * Resnik_{a,b}}{IC(a) + IC(b)} \quad \text{(S41)}$$

Used by: Zhang 2016 (BMA)[39]

Jiang and Conrath (Jiang) Similarity: Computes similarity using a distance formula based on the IC values of the MICA and terms a and b (Equation S42) [39,41].

$$Jiang_{a,b} = \frac{1}{1 + \ IC(a) + IC(b) - 2 * Resnik_{a,b}} \tag{S42}$$

Used by: Zhang 2016 (BMA)[39]

Schlicker's Relevance (Schlicker) Similarity: Computes similarity based on Lin's semantic similarity combined with a probability based on the IC value of the MICA term (Equation S43) [42].

$$Schlicker_{a,b} = Lin_{a,b} * (1 - \exp(-Rensik_{a,b})) \tag{S43}$$

Used by: Zhang 2016 (BMA)[39]

Hybrid Relative Specificity (Wu) Similarity: Computes similarity based on a combination of information content and edge distance values using the given terms and their MICA and MICD terms (Equation S44) [43].

$$Wu_{a,b} = \frac{1}{1 + Dist_{a,(MICA_{a,b})} + Dist_{b,(MICA_{a,b})}} * \frac{Resnik_{a,b}}{Rensik_{a,b} + \frac{DescIC_a + \ DescIC_b}{2}} \tag{S44}$$

Used by: Zhang 2016 (BMA)[39]

Descendent Semantic Similarities: Additionally, Zhang et. al. created versions of each of the previous 5 semantic similarities using common descendants instead of common ancestors, as shown in Equations S45–S49 [39]. **Note: We** defined these values as 0 when no common descendent is found.

$$Rensik\ Desc_{a,b} = if\left(IC(a) + IC(b) \geq IC(MICD_{a,b})\right): IC(a) + IC(b) - \ IC(MICD_{a,b}) \\ else: 1/IC(MICD_{a,b}) \tag{S45}$$

$$Lin\ Desc_{a,b} = \frac{2 * Resnik\ Desc_{a,b}}{IC(a) + IC(b)} \tag{S46}$$

$$Jiang\ Desc_{a,b} = \frac{1}{1 + \ IC(a) + IC(b) - 2 * Resnik\ Desc_{a,b}} \tag{S47}$$

$$Schlicker\ Desc_{a,b} = Lin\ Desc_{a,b} * (1 - \exp(-Rensik\ Desc_{a,b})) \tag{S48}$$

$$Wu\ Desc_{a,b} = \frac{1}{1 + Dist_{a,(MICD_{a,b})} + Dist_{b,(MICD_{a,b})}} * \frac{Resnik\ Desc_{a,b}}{Rensik\ Desc_{a,b} + \frac{Dist_{a,root} + \ Dist_{b,root}}{2}} \tag{S49}$$

All 5 Used by: Zhang 2016 (BMA)[39]

Frequency of Interaction Features:

Features in this category are created by counting the frequency annotations occur in known interactions. Given a biological concept (such as gene ontology or domain annotations), and a list of known interactions, annotation pairs are scored based on the percentage of protein pairs containing those annotations that are in the interaction list (Equation S50). The formula in Equation S50 computes the number of times each annotation pair appears in a protein pair known to interaction divided by the total number of protein pairs the term pair appears in. Since we exclude self-interactions from our dataset, we also exclude self-interacting pairs from our total count. In our experiments, we ran tests when using the full list of interactions (all), only interactions not in the test set (non-test), and only interactions whose proteins were not part of the held-out data (held out) as different experiments to test potential biasing effects.

$$Freq_{a,b} = \frac{\sum_{p1\ with\ a} \sum_{p2\ with\ b} \ (if\ ((p1, p2)\ \in interactionLst) : 1\ else\ 0)}{\sum_{p1\ with\ a} \sum_{p2\ with\ b}\ (if\ (p1\ != p2) :\ 1\ else\ 0)} \quad \text{(S50)}$$

Gene Ontology Level 2 (GO L2) PPI Frequency: GO level 2 frequency calculates the frequency pairs of GO terms 2 levels from the root of their ontology appear in interactions. Levels >= 2 were chosen to ensure the terms were not too generic. As terms in the gene ontology can have many parents, with many paths to the root, any term that was on any path that had an exact edge distance of 2 from the root was included. We computed 4 features, 1 for each of the three ontologies, and a 4th calculated using all 3 ontologies at the same time, from this computation.

Used by: Simple Ensemble (BMA)

Domain PPI Frequency: Computed from one of three different domain datasets, Pfam[44], InterPro[45], or Prosite[46]. A single frequency feature computed for each dataset used.

Used by: Zhang 2016 Domain Variant (Prod; Pfam, Prosite, InterPro) (based on study by Zhang 2016)[47], Simple Ensemble (BMA; Pfam, Prosite, InterPro)

Binary/Ternary Features:

Binary/Ternary features are created using annotations to form a binary/ternary vector of existence/non-existence for each possible annotation. The resulting feature vector is usually highly sparse. Please note, that any annotations that do not map to any proteins in our dataset our excluded when creating features.

Up to Least Common Ancestor (ULCA): Binary vector based on Semantic Similarity. For each pair of proteins, the lowest common ancestor, calculated as the common ancestor to both of protein's GO annotations with the maximal depth from the ontology root, is found. Given the lowest common ancestor, it and all descendants that are ancestors of the proteins' annotations (including the annotations themselves), are scored as 1, and all other GO terms are scored as 0. The number of features varies according to the number of annotations GO terms for a given set of annotated proteins but is around 20,000 for all 3 ontologies on the human dataset. **Note:** Maetschke's original paper states there can be multiple LCA's due to ties in depth, but did not find any clear explanation of how to handle this (whether to keep all terms, or if there is a tie-breaking procedure) [48]. We broke ties using Information Content, with ties in both Information Content and depth broken based on the order they appeared in our term list (semi-randomly). Additionally, to ensure that as many protein pairs have some data as possible, we concatenated the ULCA vectors from each of the three ontologies to create a single binary vector as a feature per protein pair.

Used by: Maetschke (2011)[48]

Pfam Domains: Binary vector based on the existence or absence of a Pfam domain being annotated to a given protein. For a pair of proteins, these vectors are combined through addition, creating a single vector where each number is either a 0, 1, or 2. The number of features varies based on the number of Pfam domains annotated in the given protein set, which is currently around 6,500 for our human dataset.

Used by: Chen 2005[49]

S4: Model Feature & Hyperparameter Processing

Our code used a total of 36 sequence, 6 non-sequence, and 5 bias based models for predicting protein interactions. In this section, we go through major possible implementation between current and previous implementations, particularly

regarding the machine learning libraries used, feature normalization, and neural network learning rate related hyperparameters. This section does not discuss any differences that may arise from feature computations, which are covered in Appendices A-C.

Machine Learning Libraries:

5 Types of machine learning models were utilized in this work, Neural Networks, Random Forest, Support Vector Machines, Rotation Forests, and Light-Gradient Boosting Machine. The following libraries were used for their calculations:

Random Forest – SciKit Learn Python Library[50]

Rotation Forest – Python Implementation of a Rotation Forest[51], implemented by Josh Loyal

Support Vector Machines – ThunderSVM, GPU version.[52]

Neural Network – PyTorch[35]

Light Gradient Boosting Machines – Python implementation of LGBM[53]

For all algorithms, we ran our implementations in a way that can produce a probability for ranking results, to ensure we can obtain metrics such as AUC. For SVMs, this involves setting the probability hyperparameter to true, forcing it to run internal cross-fold validation on the data, which may be different than previous implementations.

Scaling of Features:

When using machine learning algorithms, some models, such as random forests, are immune to scaling individual features while others, such as support vector machines (SVMs) and algorithms using Principal Component Analysis transforms can be highly sensitive to features belonging to different distributions and scales, and can generate much better computations when features are similarly scaled. While we attempted to keep most features within a similar range, we still found scaling necessary for some models to score well. For models that did not generate scores like those found in literature without scaling, or models in literature that suggested using scaling, we applied either MinMax scaling or standard scaling using the SciKit Learn preprocessing library. We kept the scaling that performed most similar on datasets studied in the previous work and used the same scaling for all datasets for a given model.

Scaling Types:

MinMax scaling scaled each feature such that the maximum value is mapped to 1, the minimum value is mapped to 0, and all other values are linearly interpolated in between.

Standard scaling normalized all feature data such that the mean is 0, and the data has a standard deviation of 1.

Given a choice between the MinMax and Standard, we preferred MinMax scaling, as the mean and variation between the features may be influenced by difference balances in positive and negative data, assuming the features generated by positive and random protein pairs contain differences in their feature distributions. MinMax should be less influenced, as the minimums and maximums given enough positive and negative training pairs should be similar.

Scaling could also be applied in two different places in the training and testing pipeline, depending on the algorithm. When using data created from protein specific features, where each protein independently generates a feature vector, scaling could be applied across all proteins in the dataset. Scaling could also be applied across all

pairs in the dataset, with the training pairs used to fit the scalers, and each individual test pair's features scaled based on those fitted values.

For each algorithm that uses scaling, the type of scaling applied is shown in Table S1.

Table S1: List of algorithms for which we added feature scaling. Feature scaling is primarily only needed when running multiple types of features in the same model and was mostly necessary only for SVM algorithms. Note that we only added feature scaling if it was necessary to make the results from previous literature and our implementations of the algorithm produce similar results.

**Model**	**Model Type**	**Protein Scaling**	**Pair Scaling**
Guo 2008[20]	SVM		Standard Scaler
Pan 2010[10]	SVM (only SVM models)		Min Max Scaler
Zhao 2012[21]	SVM		Standard Scaler
Du 2017[8]	Neural Network	Standard Scaler	
Göktepe 2018[14]	SVM	Min Max Scaler	
Tian 2019[19]	SVM		Standard Scaler

Learning Rate and Similar Neural Network Hyperparameters:

Unlike SVMs and random forests, which have generally standardized way of training models built in with the respective libraries we used, neural networks are highly sensitive to adjustments to the learning rate and the amount of data seen when training. However, while most network in literature provide a number of iterations to use when training the network, this number is for their own dataset, which may be more iterations than necessary when using larger datasets. Training a dataset 10x larger would take 10x longer, and the model would see 10x more training pairs, which may not be necessary, as the network may fit the data much quicker. To remove the limitation of using a fixed number of iterations for training, all training was done using a plateau scheduler, which adjusts the learning rate to a lower value as the network struggles to reduce training error. Additionally, all networks had a minimum learning rate set, such that, if the learning rate was reduced below the provided value prior to training the maximum number of iterations, the training would stop. This allowed us to use the same set of hyperparameters over varying sizes and distributions of data in our training sets.

We primarily relied on 6 training hyperparameters to ensure the networks ran efficiently with different datasets as shown below:

Learning Rate (LR): The rate at which the neural networks weights are updated initially, when training starts.

Minimum Learning Rate (Min LR): Minimum learning rate the algorithm runs at. When the learning rate drops below this value, the model stops training, even if it hasn't reached its maximum number of iterations.

Schedule Threshold (Thresh): The minimum amount of improvement to the loss value necessary to avoid decreasing the learning rate.

Schedule Threshold Model (Mode): Whether the schedule threshold is a percentage the best loss must decrease, or a raw value that the loss needs to improve by.

Schedule Patience (Patience): The number of epochs to wait before reducing the learning rate if the training process has not improved more than the schedule threshold.

Schedule Factor (Factor): The amount to multiply the learning rate by when the loss does not improve beyond the schedule threshold, for patience epochs.

Schedule Cooldown (Cooldown): The amount of time, in epochs, to wait between the previous learning rate decrease before considering another decrease.

The exact hyperparameters varied based on the network architecture, features used, and the optimizer used by the original implementation The hyperparameters used for each neural network algorithm are shown in Table S2.

Table S2. Hyperparameters used to adjust learning rates and converge networks early if learning has plateaued. Optimizer Type and loss type were based on parameters from original works.

Model	Loss Type	Opt Type	LR	Min LR	Thresh	Patience	Factor	Cool down	Mode
Du 2017 Sep[8]	Train	SGD	0.01	1e-2	0.01	2	0.4	0	%
Du 2017 Comb[8]	Train	SGD	0.01	1e-2	0.01	2	0.4	0	%
Sun 2017 CT Auto[11]	Train	SGD	1	1e-2	0.03	2	0.5	0	%
Sun 2017 AC Auto [11]	Train	SGD	1	1e-2	0.03	2	0.5	0	%
Gonzalez-Lopez 2018[33]	Train	RMS Prop	0.01	2e-3	0.01	2	0.5	0	abs
Hashemifar 2018 CNN[29]	Train	SGD	0.01	2e-4	0.01	3	0.4	0	%
Li 2018 CNN/LSTM[54]	Train	Adam	1e-3	2e-4	0.02	1	0.5	0	abs
Chen 2019 RNN[31]	Train	Adam	5e-4	1e-4	0.01	1, 3[a]	0.5	0	abs
Richoux 2019 LSTM[32]	Valid	Adam	1e-3	8e-4	0.01	3	0.9	0	%
Richoux 2019 Full[32]	Valid	Adam	1e-3	8e-4	0.01	3	0.9	0	%
Yao 2019 Net[55]	Train	SGD	0.01	2e-4	0.01	3	0.4	0	%
Zhang 2019 Deep[17]	Train	Adam	-[b]	-[b]	0.01	2	0.5	0	%
Li 2020 Deep[12]	Train	SGD	-[c]	1e-4	0.05	2	0.1	0	%
Czibula 2021 Auto SS[13]	Valid	Adam	0.01	1e-5	0.01	2	0.5	0	%
Czibula 2021 Auto SJ[13]	Valid	Adam	0.01	1e-5	0.01	2	0.5	0	%
Czibula 2021 Auto JJ[13]	Valid	Adam	0.01	1e-5	0.01	2	0.5	0	%
Random Network	Train	Adam	5e-4	1e-4	1.5e-3	3	0.5	0	abs

[a] Chen's implementation involved multiple LSTMs, which took time to propagate learning through. To ensure we didn't decrease the learning rate too soon, the scheduler did not start until 250,000 training pairs had been run through the model during training. A patience of 3 was used on the original, smaller data, with a patience of 1 used on our larger data, to speed up training. [b] Zhang's ensemble contained networks for each of the 3 feature types

employed, with a final ensemble network, each containing different learning rates depending on the feature type: AC lr = 7e-4, LD lr = 1e-3, MCD lr = 3e-4, Ensemble lr = 1e-3. All minimum learning rates were 1/32nd of the starting learning rates. [c] Li's model used different initial learning rates for its first layer of networks (lr = 0.2), and its final ensemble layer (lr = 0.1).

S5: List of Models

In this section, we list all models used in this work, including a short description of each model and any changes made to the models from our understanding of the original literature that are not related to feature processing, machine learning library, feature scaling, or learning rate hyperparameters used (Appendices A-D).

Sequence-Based Feature Models:

Guo 2008[20]:

Description: Uses 210 Autocovariance features per protein. Protein pairs' features are concatenated to at 420 pair feature vector, which is run through an SVM with hyperparameters C=32, gamma = 1/32.

Additional Changes: None

Pan 2010[10]:

Description: Trained 9 separate models, using 3 feature encoding schemes (Autocovariance: 210 features per protein, Conjoint Triad: 343 features per protein, PSEAAC: 40 features per protein), and 3 model types (Random Forest, Rotation Forest, SVM). Conjoint triad features were run through Latent Dirichlet Allocation, with 50 topics and alpha and beta values of 1 and 0.1 respectively. The SVM used hyperparameters of C=8, gamma =2. Features from each protein were concatenated to form protein pair feature vectors. The random forest used 500 trees, while the rotation forest used 80. Note: The AC SVM method used here and in Guo's work using an SVM is the same, expect for the scaler and hyperparameters applied.

Additional Changes: None

Zhou 2011[15]:

Description: Used 630 Local Descriptor features per protein, concatenated into 1260 features per protein pair, and trained an SVM using hyperparameters C=32, gamma =1/32.

Additional Changes: None

Zhao 2012[21]:

Description: Used a large feature set containing a variety of different sequence-based features (NMBroto, Moran, Geary, Sequence Order, Quasi, PSEAAC) to create 930 features per protein concatenated into an 1860 feature vector per protein pair used to train and test an SVM.

Additional Changes: In their original paper, Zhao et. al. utilized a grid search strategy with 5-fold validation to optimize the hyperparameters C and gamma. However, we did not find the final optimal values in their published work, and, as we tried to avoid extensive fitting for hyperparameters in our work, we utilized the default hyperparameters from our SVM library.

Jia 2015[22]:

Description: Uses 7 random forests, 1 for each of 7 different properties run through wavelet transformations, with a final vote between the forests deciding the class.

Additional Changes: To ensure the results produced were rankable (necessary for metrics such as AUC and average precision), we utilized the average score produced by the random forests as a tiebreaker for data which received the same number of positive votes.

You 2015[18]:

Description: Uses 567 MLD-based features per protein, concatenated together for each protein pair, to train a random forest with 60 trees, and a maximum of 10 features checked per split.

Additional Changes: None

Ding 2016[7]:

Description: Uses 319 features related to NMBA, AAC, and MMI calculations per protein, creating a 638 per pair feature vector used on a random forest with 500 trees, checking a maximum of 25 features per split.

Additional Changes: None

Du 2017[8]:

Description: Uses a combination of AAC, CTD, Sequence Order, Quasi, and APSEAAC features to produce 1164 features per protein. Du et. al. utilized 2 types of neural networks, one in which each protein's features are first run through an individual network before being concatenated prior to the final layers (Sep), the other concatenating a pair of proteins features prior to running the feature vector through the neural network (Comb). Both networks contain 4 hidden layers, with monotonically reducing sizes, and utilized a dropout rate of 0.2, a momentum factor of 0.9, and Xavier normal weight initialization.

Additional Changes: None

Sun 2017[11]:

Description: Created two models, one based on 210 per protein AC features, the other using 343 per protein CT features. Features were used to train a 2-layer autoencoder with 400 hidden nodes, with the second layer then removed and replaced with a single output layer. This final network is the trained to predict interactions. Both networks utilized a momentum of 0.5 when using SGD during training.

Additional Changes: We note that the original implementation was not entirely clear on how to switch from the autoencoder to predict network. Our current implementation simply swaps the second hidden layer and continues the training process with the original training hyperparameters until convergence.

Wang 2017[30]:

Description: Uses 400 PSSMDCT features, concatenated to 800 per protein pair, and a rotation forest using 5 trees and 80 features per subset.

Additional Changes: None

Göktepe 2018[14]:

Description: Computed 783 features per protein, from PSEAAC, Skip Weighted Conjoint Triad, and PSSMDPC. Each protein's feature, in each category, were run through individual Principal Component Analyses (PCA), computing 20 new features per protein per feature type. After concatenation, each protein's new 843 feature vector was run through a final PCA extractor creating a final 390 features per protein. These features were the concatenated per protein pair (780 features per pair) and used on an SVM with hyperparameters C = 32, gamma = 0.04.

Additional Changes: The size of the feature vector was noted to change from 783 to 843 after the first round of PCA. We subsequently assumed that each of the 3 PCA transformers produced 20 new features. The original paper also chose all features with an eigenvector value greater than 1, however we stuck with 390 features per final feature vector, which is the number of features that met this criteria in the initial implementation.

Gonzalez-Lopez 2018[33]:

Description: Utilized numerically encoded protein sequences as features, with protein sequences zero-padded or truncated to have a length of 1000. Truncated amino acids and padding were added or removed from the start of each protein sequence. Each protein was run through a neural network with shared parameters involving a GRU, linear layer, dropout =0.5, and 2 batch normalization layers, before the output values are concatenated to run through the remaining linear and batch norm layers and compute a final prediction.

Additional Changes: For this model, we mistakenly did not use validation data, and monitored loss exclusively off training data. Additionally, the original method utilized early stopping, rolling the network weights back to the values that maximized the results on the validation data. We simply utilized the final set of weights, which, given our strategy of reducing the learning rate to slowly converge the network, likely produced a similar result to what would have been obtained by rolling back.

Hashemifar 2018[29]:

Description: Splits PSSMs from proteins into overlapping slices of length 512, with 256 amino acids overlapping between adjacent slices. PSSMs were 0 padded from their right side (end of sequence) to ensure their lengths were multiples of 256. During testing, each pair of proteins is assigned the maximal score provided from any pair of slices. The neural network contained a shared architecture utilized by both proteins individually, containing multiple convolutions, pooling, and batch norm layers. Once run through the shared architecture, proteins were run through random project, an unshared batch normalization layer, and multiplied together through element-wise multiplication prior to running through a single linear layer and outputting a prediction. The SGD optimizer used by the algorithm had a momentum factor of 0.9, and weights were initialized using a normal distribution.

Additional Changes: None

Li 2018[54]:

Description: Used numerically encoded protein sequences, 0 padded or truncated from their left (start of sequence) to be length 1200 as features. Each protein's feature vector was run through a shared parameter network consisting of multiple convolution and pooling layers and a single LSTM, before concatenating prior to a final linear layer.

Additional Changes: In the original implementation, proteins with a length greater than 1200 were removed. To keep the feature set similar across different models, we used truncated, which is used by other algorithm in literature, to reduce proteins' lengths down to 1200 amino acids.

Chen 2019 LGBM[16]:

Description: Creates per protein feature vectors from NMBA, Moran, Geary, PSEAAC, Local Descriptor, and Conjoint Triad techniques to create 1185 features per protein. After concatenating for each pair, Elastic net is used to reduce the number of features, prior to passing the features into an LGBM classifier. Hyperparameters of alpha=0.01, and l1ratio = 0.1 are used on the elastic net, and features obtaining a non-zero network coefficient were kept. The number of boosted trees in the classifier was set to 500.

Additional Changes: None

Chen 2019 RNN[31]:

Description: Uses a combination of skip gram and one hot encoding to create 12 features per amino acid per protein sequence. Proteins were zero padded or truncated on their right side (end of sequence) to be of length 2000, and each protein's features were run through a parameter shared neural network consisting of 5-6 convolution, GRU, and pooling layers, before concatenating together and running through a final set of linear layers.

Additional Changes: The original paper suggested encoding to the size of the longest protein. However, the value 2000 was used in the source code provided by the authors regardless of sequence length, and subsequently used by our method, with long protein sequences truncated.

Jia 2019[9]:

Description: Generates 36 per protein features from Chaos and AAC, concatenates features per protein pair, and uses a random forest, with 200 trees, to predict interactions.

Additional Changes: None

Richoux 2019[32]:

Description: Utilized one-hot encoding per amino acid, with a maximum protein length of 1166, truncating or zero padding from the right side (end of sequence) of the protein sequence. Protein features were run though a shared network, of either multiple linear and batch norm layers (Full network) or multiple convolutions, batch norms, and an LSTM (LSTM network), prior to concatenating and running through a final set of batch norm and linear layers for predictions. 10% of the training data was held out as validation data, to use to compute the network's loss and prevent overfitting. Weights were initialized using Xavier Uniform.

Additional Changes: None

Tian 2019[19]:

Description: Used EGBW, AC, and PSEAAC features to compute 139 per protein features, concatenated into 278 features per protein pair. Features were run through 2D-denoising wavelets with hard thresholding and the db8 wavelet algorithm, before being used to predict protein interactions using an SVM.

Additional Changes: The original implementation used max lag values of 9-11 for different physicochemical property-based features, due in part of the length of the smallest sequence in one of their datasets being length 12. Despite filtering out small sequences for our testing purposes, we did not change these hyperparameters.

The processing method run for this algorithm, 2D-denoising wavelets, runs on 2-dimensional data. To the best of our knowledge, the authors created this data by stacking features vectors for all protein pairs into a single 2D matrix, thus creating an order dependency between the data. The denoising wavelets, which are designed to remove artifacts in images, subsequently remove differences between values in different rows, ensuring protein pairs 1 and 2 were more similar if they were ordered above and below one another, even though the data should be order dependent. This alone wouldn't boost the results, except for 2 other conditions that we believe were also met in the original implementation. First, the data appears to have been sorted by class, which allows the denoising the make feature vectors for positive data more similar, and feature vectors for negative data more similar. Secondly, cross-validation was not performed until after the denoising step, meaning that all positive class feature vectors for training and test data were made more similar, and all negative class feature vectors for training and test data were made more similar, prior to running cross-validation. Under this preprocessing methodology, increasing the threshold of the denoising wavelet (which controls how similar to make adjacent data) can trivially achieve over 98% accuracy on almost any dataset. In a real-world application, the class of all data cannot be known prior to testing, otherwise there would be no use for the model. For our implementation, we split the data into training and test sets and shuffled the data, prior to running denoising, and found the results tended to be worse than running without using denoising on the feature vectors.

Yao 2019[55]:

Description: Uses 20 features per amino acid calculated from a skip-gram model, with a maximum protein length of 850, zero padding or truncating values from the right side (end of sequence) of each amino acid sequence. Each protein per protein pair was run through its own independent network consisting of batch norm, linear, and dropout layers (with dropout = 0.5), prior to concatenating for a final set of linear and batch norm layers to predict an interaction.

Additional Changes: The original paper suggests zero padding for sequences less than 850 but does not clearly specify what to do for proteins over a length of 850. We truncated, as done in other papers.

Zhang 2019[17]:

Description: Using 210 per protein features from AC, 630 per protein features from LD, and 1449 per protein features from MCD, Zhang et. al. generated an ensemble of 27 neural networks, 9 per features, with a final 28th network used to predict interactions from the output of the first 27 networks. All 28 networks consisted primarily of linear and dropout layers, with concatenated protein pair data used as the input to the first 27 networks. Weights for each network were initialized using Xavier Normal.

Additional Changes: While the original paper provided a set of recommended depths, widths, and dropout rates to generate 9 networks per feature type, the exact combination of these properties used in the paper is not listed. While we used values from these lists to generate networks, we cannot guarantee the exact hyperparameters per network were used in the original implementation.

Li 2020[12]:

Description: Using 210 per protein features from AC, 35 per protein features from PSEAAC, 343 per protein features from conjoint triad, and 630 per protein features from LD, generated an ensemble neural networks with a final prediction network utilizing outputs from the first layer of networks. Each network involves per protein processing steps through linear and convolutions layer(s), prior to running the proteins through a multiheaded attention module, running pooling layers, and concatenating the resulting protein features before a final set of linear and batch norm layers.

Each feature utilizes 4 different network structures, with 3 subsets of training data, creating 12 first layer networks per features type, or 48 first layer networks total. Each subset relies on holding out 33% of the training data, which is used to compute values to pass to train the final layer. Through this method, each training pair is used for training 1 times, and thus each first layer network only produces outputs for 1/3 of all training data. To match this during the testing process, the values output by the 12 networks are averaged into 4 values, with the outputs of networks using the same hyperparameters but different subsets of training data being averaged together.

Additional Changes: To simplify the run time (and because it didn't appear to negatively affect the result), we reduced from 5 folds to 3 folds in the network construction phase, reducing the total number of networks to 49 (48 first layer, 1 final layer) from the originally used 81 networks (80 first layer, 1 final layer). We also multiplied PSEAAC values by 100, as this appeared to help the network fit to them better.

In terms of the network structure, we did not find any explicitly mention of what makes the 4 different models per feature type different in the original paper. For our implementation, we modified the number of convolution layers applied, the number of heads in the multi-headed attention layer, and the number of linear layers at the end of each network to make different network structures per feature. However, we note this is likely different than the original implementation.

Czibula 2021[13]:

Description: Used 420 features per protein from AC, and 343 features per protein from conjoint triad methods, to create 763 per protein features. Features were run through autoencoders, which either concatenated the protein pairs and ran a single pass through the encoder and decoder (JJ), ran each protein through a shared set of weights for the encoder, before concatenating and running through a decoder (SJ), or ran each protein through a shared set of weights on the encoder and decoder, concatenating each protein with an additional vector consisting of the product of both protein encoded features between the encoder and decoder networks (SS). Each encoder and decoder consisted of 3 linear layers. For each type of network, 2 autoencoders were trained, 1 exclusively on positive data, and 1 exclusively on negative data. To predict interactions, the average per feature loss from the positive and negative autoencoders was compared, with the classes used to train the network with the lowest average loss being chosen as the predicted class. During training, 10% data was held out for validation.

Additional Changes: None

Non-Sequence Based Feature Models

Chen 2005[49]:

Description: Represents proteins pairs as ternary valued vectors, created from the sum of binary vectors of the existence of absence of a Pfam domain. Predictions are made using a random forest with 150 trees, with a minimum of 3 pairs remaining per split and a maximum depth of 450 per tree.

Additional Changes: The original implementation mentions an impurity hyperparameter of 0.01. However, this did not work with the min impurity decrease hyperparameter provided by the SciKit Learn random forest, causing the forest to not learn from the training data, and was subsequently removed from our code.

Guo 2006[38]:

Description: Creates features per protein pair using semantic similarity with maximum aggregation. A logistic regression model is used to predict interactions from the set of 3 similarity features.

Additional Changes: None

Maetschke 2012[48]:

Description: Utilized up to the lowest common ancestor as features per protein pair and predicted interactions using a random forest with 200 trees, with each split checking a maximum of 200 features.

Additional Changes: None

Zhang 2016[39]:

Description: Computes 30 semantic similarity values from 10 different calculations on ascending and descending similarities using BMA aggregation, and predicts interactions using an SVM.

Additional Changes: Original paper relied on computing hyper parameters for C and gamma using a grid search method. As we are not focused on optimizing hyperparameters per dataset, we simply left the hyperparameters as their default values provided by our SVM library.

Domain Variant:

Description: Computed the probability of a pair of domains interacting aggregated using maximum aggregation and predicted interactions using logistic regression.

Additional Changes: None. This is a novel protein interaction prediction method, based loosely on Zhang 2016 Dom [47].

Simple Ensemble:

Description: Computes protein interactions based on features computed from Resnik semantic similarity, and the probability of level 2 GO terms and domains existing in interactions. Predictions are done on a random forest with 30 trees.

Additional Changes: None. This is a novel, simplistic predictor loosely inspired by previous works from Thahir et. al. [56] and Qi et. al. [4].

Bias Predictors:

Count Bias:

Description: Predicts interactions by counting the number of training examples containing each protein that were positive and negative.

Additional Changes: None, novel.

Seq Sim Bias:

Description: Predicts interactions by counting the number of positive and negative training examples containing the up to 5 most sequence similar proteins in the training set to each protein in the test pair. The most sequence similar proteins are weighted by their sequence similarity.

Additional Changes: None, novel.

Seq Sim + Protein Bias:

Description: Predicts interactions by counting the number of positive and negative training examples containing the up to 5 most similar genes based on whether the genes share proteins and sequence similarity. The most similar proteins are weighted by their similarity.

Additional Changes: None, novel.

Rand Net:

Description: Predicts interactions using 500 random number features per protein, with each protein running through a parameter shared network of linear and dropout layers prior to concatenating before a final linear layer. Shared features per protein were chosen over concatenating proteins prior to starting the neural network as this allows the network to learn each protein easier, making good predictions based on simple biases more likely.

Additional Changes: None, novel.

Rand RF:

Description: Predicts interactions using 500 random number features per protein, using default hyperparameters for the random forest.

Additional Changes: None, novel.

S6: Bias/Illogical/Counting Features

Features, such as sequence similarity and counting proteins in training data used to compute bias/illogical feature based calculations.

Count Bias: Count bias simply computes, for each protein in the training set, the number of times the protein appears in interacting and non-interacting pairs, adding +1 for interacting appearances and -1 for non-interacting appearances. For a given pair of proteins, their values are added together, with the result being the final prediction score assigned to the protein pair.

Used by: Count Bias

Sequence Similarity (Seq Sim) Bias: Sequence similarity bias calculates the similarity of all sequences in the protein set and maps each protein in the test dataset to its 5 nearest neighbors in the training set. Only proteins that appear at least once in the training set are considered as possible nearest neighbors. Similarity is computed based on the e-values return by as BLAST query between the proteins. If less than 5 proteins met the minimum similarity threshold e-value = 10, then only the neighbors that meet the criteria are used. E-values are converted into similarity score values using Equation S51, which maps the e-values to a 0-1 range. Given the similarity

values of the up to 5 nearest neighbors, these weight values are normalized to sum to 1, and the final score for each protein is the weighted sum of the 5 nearest neighbors multiplied by their count bias, as shown in Equation S52.

$$SimScore_{eval} = \min\big(abs(log_{10}(E - Value)\big), 200)/200 \quad \text{(S51)}$$

$$SeqSimBias_{(neighbors,weights)} = \sum_{n,w\,\in(neighbors,weights)} w * Count\ Bias_n \quad \text{(S52)}$$

Used by: Seq Sim Bias

Sequence Similarity Bias + Protein Bias (Seq Sim + Prot Bias): The sequence similarity bias + protein bias is calculated the same as the sequence similarity bias but adds to the SimScore whenever two genes share the same protein. The Protein Bias is computed as the number of proteins shared between both proteins, divided by the number of proteins total between the two genes, with an added weight factor, as defined in Equation S53. This bias is then added to the SimScore as shown in Equation S54. The final normalization and computation is done in the same manner the sequence similarity bias is compute (final computation shown in Equation S52. For our calculations, the bonus value w was set as 1.

$$ProtSim_{a,b} = \frac{Intersection(proteins\ \in\ A,\ proteins\ \in\ B)}{Union(proteins\ \in\ A,\ proteins\ \in B)} \quad \text{(S53)}$$

$$SimScore + Prot_{a,b} = SimScore_{E-vValue_{a,b}} + (ProSim_{a,b} + (w\ if\ ProtSim_{a,b} > 0)) \quad \text{(S54)}$$

Used by: Seq Sim + Prot Bias

Random Numbers: Random numbers are generated by the PyTorch library. For our computations, we drew 500 random numbers per protein ranging between 0 and 1.

Used by: Rand Net, Rand RF

S7: Notes on Evaluation Metrics

Accuracy values are taken at the threshold on the ROC curve which maximizes the accuracy, per test. AUC is computed used SciKit-Learn's built in AUC function [50]. Average precision is computed as the average of the precision values at each unique recall value (x-axis) multiplied by the distance in recall (x-axis) since the previous value.

Calculations done in tables are averaged across all datasets utilized for a given test. For example, Precision at 3% recall values are based on the average across all datasets at 3% recall for the given model and set of test datasets. Plots are based on a concatenation of all data, which may generate slightly different results, but should be representative of the averages taken.

References

1. Stark, C.; Breitkreutz, B.-J.; Reguly, T.; Boucher, L.; Breitkreutz, A.; Tyers, M. BioGRID: a general repository for interaction datasets. *Nucleic Acids Res.* **2006**, *34*, D535–D539.
2. National Center for Biotechnology Information. Available online: https://www.ncbi.nlm.nih.gov/ (accessed on Feb/May, 2021).
3. Ganapathiraju, M.K.; Thahir, M.; Handen, A.; Sarkar, S.N.; Sweet, R.A.; Nimgaonkar, V.L.; Loscher, C.E.; Bauer, E.M.; Chaparala, S. Schizophrenia interactome with 504 novel protein–protein interactions. *NPJ schizophrenia* **2016**, *2*, 1–10.

4. Qi, Y.; Dhiman, H.K.; Bhola, N.; Budyak, I.; Kar, S.; Man, D.; Dutta, A.; Tirupula, K.; Carr, B.I.; Grandis, J. Systematic prediction of human membrane receptor interactions. *Proteomics* **2009**, *9*, 5243–5255.
5. Li, Y.; Ilie, L. SPRINT: ultrafast protein-protein interaction prediction of the entire human interactome. *BMC Bioinform.* **2017**, *18*, 485, doi:10.1186/s12859-017-1871-x.
6. Keshava Prasad, T.t.; Goel, R.; Kandasamy, K.; Keerthikumar, S.; Kumar, S.; Mathivanan, S.; Telikicherla, D.; Raju, R.; Shafreen, B.; Venugopal, A. Human protein reference database—2009 update. *Nucleic Acids Res.* **2009**, *37*, D767-D772.
7. Ding, Y.; Tang, J.; Guo, F. Predicting protein-protein interactions via multivariate mutual information of protein sequences. *BMC Bioinform.* **2016**, *17*, 1–13.
8. Du, X.; Sun, S.; Hu, C.; Yao, Y.; Yan, Y.; Zhang, Y. DeepPPI: boosting prediction of protein–protein interactions with deep neural networks. *J. Chem. Inf. Model.* **2017**, *57*, 1499–1510.
9. Jia, J.; Li, X.; Qiu, W.; Xiao, X.; Chou, K.-C. iPPI-PseAAC (CGR): Identify protein-protein interactions by incorporating chaos game representation into PseAAC. *J. Theor. Biol.* **2019**, *460*, 195–203.
10. Pan, X.-Y.; Zhang, Y.-N.; Shen, H.-B. Large-Scale prediction of human protein− protein interactions from amino acid sequence based on latent topic features. *J. Proteome Res* **2010**, *9*, 4992–5001.
11. Sun, T.; Zhou, B.; Lai, L.; Pei, J. Sequence-based prediction of protein protein interaction using a deep-learning algorithm. *BMC Bioinform* **2017**, *18*, 1–8.
12. Li, F.; Zhu, F.; Ling, X.; Liu, Q. Protein Interaction Network Reconstruction Through Ensemble Deep Learning With Attention Mechanism. *Front. Bioeng. Biotechnol* **2020**, *8*, 390.
13. Czibula, G.; Albu, A.-I.; Bocicor, M.I.; Chira, C. AutoPPI: An Ensemble of Deep Autoencoders for Protein–Protein Interaction Prediction. *Entropy* **2021**, *23*, 643.
14. Göktepe, Y.E.; Kodaz, H. Prediction of protein-protein interactions using an effective sequence based combined method. *Neurocomputing* **2018**, *303*, 68–74.
15. Zhou, Y.Z.; Gao, Y.; Zheng, Y.Y. Prediction of protein-protein interactions using local description of amino acid sequence. In *Advances in computer science and education applications*; Springer: Qingdao, China, 2011; pp. 254–262.
16. Chen, C.; Zhang, Q.; Ma, Q.; Yu, B. LightGBM-PPI: Predicting protein-protein interactions through LightGBM with multi-information fusion. *Chemom. Intell. Lab. Syst.* **2019**, *191*, 54–64.
17. Zhang, L.; Yu, G.; Xia, D.; Wang, J. Protein–protein interactions prediction based on ensemble deep neural networks. *Neurocomputing* **2019**, *324*, 10–19.
18. You, Z.-H.; Chan, K.C.; Hu, P. Predicting protein-protein interactions from primary protein sequences using a novel multi-scale local feature representation scheme and the random forest. *PLoS ONE* **2015**, *10*, e0125811.
19. Tian, B.; Wu, X.; Chen, C.; Qiu, W.; Ma, Q.; Yu, B. Predicting protein–protein interactions by fusing various Chou's pseudo components and using wavelet denoising approach. *J. Theor. Biol.* **2019**, *462*, 329–346.
20. Guo, Y.; Yu, L.; Wen, Z.; Li, M. Using support vector machine combined with auto covariance to predict protein–protein interactions from protein sequences. *Nucleic Acids Res.* **2008**, *36*, 3025–3030.
21. Zhao, X.-W.; Ma, Z.-Q.; Yin, M.-H. Predicting protein-protein interactions by combing various sequence-derived features into the general form of Chou's Pseudo amino acid composition. *Protein Pept. Lett* **2012**, *19*, 492–500.
22. Jia, J.; Liu, Z.; Xiao, X.; Liu, B.; Chou, K.-C. iPPI-Esml: an ensemble classifier for identifying the interactions of proteins by incorporating their physicochemical properties and wavelet transforms into PseAAC. *J. Theor. Biol.* **2015**, *377*, 47–56.
23. Chou, K.-C. Prediction of protein subcellular locations by incorporating quasi-sequence-order effect. *Biochem. Biophys. Res. Commun.* **2000**, *278*, 477–483.
24. Schneider, G.; Wrede, P. The rational design of amino acid sequences by artificial neural networks and simulated molecular evolution: de novo design of an idealized leader peptidase cleavage site. *Biophysical Journal* **1994**, *66*, 335–344.
25. Grantham, R. Amino acid difference formula to help explain protein evolution. *Science* **1974**, *185*, 862–864.
26. Chou, K.-C. Using amphiphilic pseudo amino acid composition to predict enzyme subfamily classes. *Bioinformatics* **2005**, *21*, 10–19.
27. Altschul, S.F.; Madden, T.L.; Schäffer, A.A.; Zhang, J.; Zhang, Z.; Miller, W.; Lipman, D.J. Gapped BLAST and PSI-BLAST: a new generation of protein database search programs. *Nucleic Acids Res.* **1997**, *25*, 3389–3402.
28. UniProt: the universal protein knowledgebase in 2021. *Nucleic Acids Res.* **2021**, *49*, D480–D489.
29. Hashemifar, S.; Neyshabur, B.; Khan, A.A.; Xu, J. Predicting protein–protein interactions through sequence-based deep learning. *Bioinformatics* **2018**, *34*, i802–i810.
30. Wang, L.; You, Z.-H.; Xia, S.-X.; Liu, F.; Chen, X.; Yan, X.; Zhou, Y. Advancing the prediction accuracy of protein-protein interactions by utilizing evolutionary information from position-specific scoring matrix and ensemble classifier. *J. Theor. Biol.* **2017**, *418*, 105–110.
31. Chen, M.; Ju, C.J.-T.; Zhou, G.; Chen, X.; Zhang, T.; Chang, K.-W.; Zaniolo, C.; Wang, W. Multifaceted protein–protein interaction prediction based on Siamese residual RCNN. *Bioinformatics* **2019**, *35*, i305–i314.
32. Richoux, F.; Servantie, C.; Borès, C.; Téletchéa, S. Comparing two deep learning sequence-based models for protein-protein interaction prediction. *Arxiv preprint arXiv* 2019, *1901.06268*.

33. Gonzalez-Lopez, F.; Morales-Cordovilla, J.A.; Villegas-Morcillo, A.; Gomez, A.M.; Sanchez, V. End-to-end prediction of protein-protein interaction based on embedding and recurrent neural networks. In Proceedings of the 2018 IEEE International Conference on Bioinformatics and Biomedicine (BIBM), Madrid, Spain, 3–6 December 2018, 2018; pp. 2344–2350.
34. Mikolov, T.; Chen, K.; Corrado, G.; Dean, J. Efficient estimation of word representations in vector space. *arXiv preprint arXiv* **2013**, 1301.3781.
35. Paszke, A.; Gross, S.; Massa, F.; Lerer, A.; Bradbury, J.; Chanan, G.; Killeen, T.; Lin, Z.; Gimelshein, N.; Antiga, L. Pytorch: An imperative style, high-performance deep learning library. *Adv. Neural Inf. Process. Syst.* **2019**, *32*, 8026–8037.
36. Klopfenstein, D.; Zhang, L.; Pedersen, B.S.; Ramírez, F.; Vesztrocy, A.W.; Naldi, A.; Mungall, C.J.; Yunes, J.M.; Botvinnik, O.; Weigel, M. GOATOOLS: A Python library for Gene Ontology analyses. *Scientific reports* **2018**, *8*, 1–17.
37. Resnik, P. Using information content to evaluate semantic similarity in a taxonomy. *arXiv preprint cmp-lg/9511007* **1995**.
38. Guo, X.; Liu, R.; Shriver, C.D.; Hu, H.; Liebman, M.N. Assessing semantic similarity measures for the characterization of human regulatory pathways. *Bioinformatics* **2006**, *22*, 967–973.
39. Zhang, S.-B.; Tang, Q.-R. Protein–protein interaction inference based on semantic similarity of gene ontology terms. *Journal of theoretical biology* **2016**, *401*, 30-37.
40. Lin, D. An information-theoretic definition of similarity. *Icml* **1998**, *98*, 296–304.
41. Jiang, J.J.; Conrath, D.W. Semantic similarity based on corpus statistics and lexical taxonomy. *arXiv preprint cmp-lg/9709008* **1997**.
42. Schlicker, A.; Domingues, F.S.; Rahnenführer, J.; Lengauer, T. A new measure for functional similarity of gene products based on Gene Ontology. *BMC bioinformatics* **2006**, *7*, 1–16.
43. Wu, X.; Pang, E.; Lin, K.; Pei, Z.-M. Improving the measurement of semantic similarity between gene ontology terms and gene products: insights from an edge-and IC-based hybrid method. *PloS one* **2013**, *8*, e66745.
44. Bateman, A.; Coin, L.; Durbin, R.; Finn, R.D.; Hollich, V.; Griffiths-Jones, S.; Khanna, A.; Marshall, M.; Moxon, S.; Sonnhammer, E.L. The Pfam protein families database. *Nucleic Acids Res.* **2004**, *32*, D138–D141.
45. Hunter, S.; Apweiler, R.; Attwood, T.K.; Bairoch, A.; Bateman, A.; Binns, D.; Bork, P.; Das, U.; Daugherty, L.; Duquenne, L. InterPro: the integrative protein signature database. *Nucleic Acids Res.* **2009**, *37*, D211–D215.
46. Hulo, N.; Bairoch, A.; Bulliard, V.; Cerutti, L.; De Castro, E.; Langendijk-Genevaux, P.S.; Pagni, M.; Sigrist, C.J. The PROSITE database. *Nucleic Acids Res.* **2006**, *34*, D227–D230.
47. Zhang, X.; Jiao, X.; Song, J.; Chang, S. Prediction of human protein–protein interaction by a domain-based approach. *J. Theor. Biol.* **2016**, *396*, 144–153.
48. Maetschke, S.R.; Simonsen, M.; Davis, M.J.; Ragan, M.A. Gene Ontology-driven inference of protein–protein interactions using inducers. *Bioinformatics* **2012**, *28*, 69–75.
49. Chen, X.-W.; Liu, M. Prediction of protein–protein interactions using random decision forest framework. *Bioinformatics* **2005**, *21*, 4394–4400.
50. Pedregosa, F.; Varoquaux, G.; Gramfort, A.; Michel, V.; Thirion, B.; Grisel, O.; Blondel, M.; Prettenhofer, P.; Weiss, R.; Dubourg, V. Scikit-learn: Machine learning in Python. *J. Mach. Learn. Res.* **2011**, *12*, 2825–2830.
51. Rodriguez, J.J.; Kuncheva, L.I.; Alonso, C.J. Rotation forest: A new classifier ensemble method. *IEEE Trans. Pattern Anal. Mach. Intell.* **2006**, *28*, 1619–1630.
52. Wen, Z.; Shi, J.; Li, Q.; He, B.; Chen, J. ThunderSVM: A fast SVM library on GPUs and CPUs. *J. Mach. Learn. Res.* **2018**, *19*, 797–801.
53. Ke, G.; Meng, Q.; Finley, T.; Wang, T.; Chen, W.; Ma, W.; Ye, Q.; Liu, T.-Y. Lightgbm: A highly efficient gradient boosting decision tree. *Adv. Neural Inf. Process. Syst.* **2017**, *30*, 3146–3154.
54. Li, H.; Gong, X.-J.; Yu, H.; Zhou, C. Deep neural network based predictions of protein interactions using primary sequences. *Molecules* **2018**, *23*, 1923.
55. Yao, Y.; Du, X.; Diao, Y.; Zhu, H. An integration of deep learning with feature embedding for protein–protein interaction prediction. *PeerJ* **2019**, *7*, e7126.
56. Thahir, M.; Sharma, T.; Ganapathiraju, M.K. An efficient heuristic method for active feature acquisition and its application to protein-protein interaction prediction. In Proceedings of BMC proceedings; pp. 1–9.
